# Upcycling of Citrus Waste by Natural Deep Eutectic Solvents: Green Extraction of Bioactive Compounds with Antioxidant and Regenerative Properties on Human Keratinocytes

**DOI:** 10.3390/nu17233692

**Published:** 2025-11-25

**Authors:** Alessia Silla, Angela Punzo, Rossana Comito, Emanuele Porru, Greta Gozzi, Maria Cristina Barbalace, Matteo Perillo, Antonello Lorenzini, Marco Malaguti, Silvana Hrelia, Cristiana Caliceti

**Affiliations:** 1 Department of Biomedical and Neuromotor Sciences, Alma Mater Studiorum, University of Bologna, 40126 Bologna, Italy; alessia.silla2@unibo.it (A.S.); angela.punzo2@unibo.it (A.P.); matteo.perillo2@unibo.it (M.P.); antonello.lorenzini@unibo.it (A.L.); 2 Biostructures and Biosystems National Institute (INBB), 00185 Rome, Italy; emanuele.porru2@unibo.it; 3 Occupational Medicine Unit, Department of Medical and Surgical Sciences, Alma Mater Studiorum, University of Bologna, 40138 Bologna, Italy; rossana.comito2@unibo.it; 4 Department for Life Quality Studies, Alma Mater Studiorum, University of Bologna, 47921 Rimini, Italy; maria.barbalace2@unibo.it (M.C.B.); marco.malaguti@unibo.it (M.M.); silvana.hrelia@unibo.it (S.H.); 5 Interdepartmental Centre for Renewable Sources, Environment, Sea and Energy (CIRI FRAME), Alma Mater Studiorum, University of Bologna, 40126 Bologna, Italy; 6 Interdepartmental Centre for Industrial Agrofood Research-CIRI Agrofood, University of Bologna, 47521 Cesena, Italy

**Keywords:** citrus waste, natural deep eutectic solvents (NaDES), antioxidant activity, wound healing, keratinocytes, upcycling, cosmeceuticals

## Abstract

**Background:** The citrus processing industry generates over 40 million tons of waste annually, representing a significant environmental challenge. Citrus by-products are rich in bioactive compounds with proven health benefits. This study aims to upcycle citrus waste by developing green extracts and evaluating their biological activities for cosmeceutical applications. **Methods:** Three NaDES formulations—choline chloride–urea (ChCl: U), choline chloride–citric acid (ChCl: CA), and betaine–urea (Bet: U)—were optimized to extract polyphenols from orange and lemon waste using roller agitation. Extracts were characterized by HPLC–ESI–MS/MS. Biological activities were assessed in human keratinocytes (HaCaT). Antioxidant activity was measured using a chemiluminescent assay that detects intracellular H_2_O_2_ production. The wound-healing potential was evaluated using scratch assays, and cytokine release (IL-6, IL-8, IL-1β, IL-10) was assessed by ELISA. DNA damage protection was evaluated by quantifying 53BP1 foci following genotoxic exposure (neocarzinostatin). **Results**: All NaDES extracts showed high polyphenol content, with hesperidin being the primary compound. Pretreatment with the extracts for 24 h significantly reduced intracellular H_2_O_2_ levels, confirming their antioxidant efficacy. In scratch assays, extracts enhanced wound closure; notably, the Bet: U-derived orange extract achieved complete closure within 48 h. All extracts increased IL-6 and IL-8 release, consistent with an early pro-regenerative response. Pretreatment with the Bet: U orange extract lowered the number of cells with high 53BP1 foci after genotoxic stress, indicating partial DNA damage protection. **Conclusions:** These findings highlight citrus by-product extracts as sustainable bioactive ingredients with great potential for skin repair and anti-aging formulations, promoting responsible cosmeceutical innovation.

## 1. Introduction

Citrus fruits, comprising various species within the genus *Citrus* (family *Rutaceae*), constitute one of the most extensively cultivated and economically significant crop groups globally. Renowned for their sensory qualities and rich nutritional profile, which includes vitamin C, dietary fiber, and phytochemicals, citrus fruits are widely linked to enhanced immune function, improved cardiovascular health, and reduced incidence of chronic diseases [1,2]. Global citrus production is estimated at approximately 22.5 million tonnes for the 2024/2025 season, with the Mediterranean region leading in the cultivation of oranges and lemons [3].

More than 40% of the world’s citrus fruit production is processed into various products, including preserves, fresh juices, and flavorings [4]. This process yields significant quantities of agro-industrial residues, primarily comprising peels, seeds, and residual pulp, which account for up to 50% of the initial fruit mass [5]. The high organic content of this waste poses challenges for direct disposal as it can disrupt soil and water quality, highlighting the urgent need for sustainable valorization strategies.

Recent studies have emphasized the potential of citrus by-products as an excellent source of bioactive compounds suitable for applications in the nutraceutical and cosmeceutical fields [6,7]. Citrus peels and seeds contain significantly higher concentrations of polyphenols and specific flavonoids, including narirutin, hesperidin, and naringin, than the edible pulp [8]. These phytochemicals have demonstrated a broad spectrum of biological activities that benefit human health [2,9].

Upcycling agricultural waste in cosmeceutical formulations represents a promising example of circular economy principles [10]. Despite the well-documented bioactive profile of citrus waste, the use of these secondary streams for high-value applications remains limited. Addressing this gap through innovative technologies and green solvents can simultaneously reduce environmental impact and create new opportunities in the cosmeceutical market, thereby supporting sustainable development. In this context, natural deep eutectic solvents (NaDESs) have emerged as environmentally friendly and effective solvents for recovering valuable compounds [11]. These methodologies align with circular economy principles and greatly decrease the environmental impacts of traditional solvents.

Skin aging is a complex and multifactorial phenomenon marked by structural degradation, reduced barrier function, and impaired regenerative capacity, primarily driven by oxidative stress. Reactive oxygen species (ROS) contribute to extracellular matrix degradation, cellular senescence, and diminished activity of fibroblasts and keratinocytes, ultimately leading to compromised collagen synthesis and delayed wound healing [12]. Therefore, phytochemicals that can mitigate oxidative stress and support skin function are appealing candidates for cosmetic applications.

This study aims to investigate the biological properties of citrus waste-derived extracts obtained through NaDES-based green extraction, to develop sustainable, high-value ingredients for topical formulations. By upcycling citrus by-products, this research supports innovation in skin health and advances the circular cosmeceutical industry.

## 2. Materials and Methods

### 2.1. Chemicals

Phosphate-buffered saline (PBS) tabs (giving a 137 mM NaCl, 2.7 mM KCl phosphate-buffered solution, pH 7.4, final concentration 0.01 M), trypsin–EDTA, the antibiotic solution 100× (10,000 U/mL penicillin and 10 mg/mL streptomycin), Gallic acid, the Folin–Ciocalteu phenol reagent, Hydroxytyrosol, Fluorescein, 2,2′-Azobis(2-methylpropionamidine) dihydrochloride (AAPH), Trolox, neocarzinostatin, Tween 20, bovine serum albumin (BSA), ethanol and the oxidative stress inducers lipopolysaccharide (LPS) and menadione were obtained from Sigma-Aldrich (St. Louis, MO, USA). An LPS stock solution (5 mg/mL) was prepared in PBS and stored at −20 °C. A menadione stock solution (100 mM) was prepared in DMSO and stored at −20 °C. Choline chloride (ChCl, ≥98%), Betaine (Bet, ≥98%), Urea (U, ≥99%), DL-malic acid (MA, ≥98%), Citric Acid (CA, ≥98%), L-(+)-Tartaric acid (TA, ≥99.5%), and D-(+)-Glucose (Glu, ≥99.5%) were obtained from Sigma-Aldrich (St. Louis, MO, USA). Dulbecco’s Modified Eagle Medium (DMEM) high glucose was acquired from Microgem (Naples, Italy), and fetal bovine serum (FBS) was acquired from Thermo Fisher Scientific (Waltham, MA, USA). The WST-8 and Cytotoxicity Lactate Dehydrogenase (LDH) Assay kits were acquired from Dojindo Molecular Technologies (Kumamoto, Japan). The chemiluminescent probe (AquaSpark™510 Peroxide Probe) was provided by Biosynth Carbosynth (Staad, Switzerland). A 10 mM mother solution was prepared by dissolving the probe in DMSO. A RNeasy Mini Kit was purchased from QIAGEN (Hilden, Germany). Primers for Real-Time PCR were purchased from IDT (Coralville, IA, USA). Analytical standards of hesperidin, hesperetin, naringin, and rutin were purchased from Sigma-Aldrich (St. Louis, MO, USA). Isotopically labeled standards rutin-D_3_ were obtained from Santa Cruz Biotechnology (Dallas, TX, USA), hesperetin-^13^C-D_4_ from Sigma-Aldrich (St. Louis, MO, USA), and naringenin-D_4_ from MedChemExpress (Monmouth Junction, NJ, USA).

### 2.2. Raw Material

Oranges (*Citrus sinensis*) and lemon (*Citrus limon*) pomace, including peels and seeds, were kindly provided by Dr. Paolo Rapisarda (Acireale, CT, Italy). After collection, the pomace was freeze-dried for 24 h using a Labconco™ FreeZone™ (Labconco Corporation, Kansas City, MO, USA) 4.5 L and stored at −80 °C until the experiments were conducted.

### 2.3. Preparation and Characterization of NaDES Mixtures

NaDESs were prepared according to the methodology established by Dai et al. [13], utilizing the molar ratios specified in Table 1.

Briefly, Choline Chloride (ChCl) and Betaine (Bet) served as hydrogen bond acceptors (HBAs), while Urea (U), Glucose (G), Malic acid (MA), Citric acid (CA), and Tartaric acid (TA) acted as hydrogen bond donors (HBDs). The two components of the mixture were combined within a container, subjected to magnetic stirring, and heated to 70 °C until a transparent liquid was obtained. Subsequently, distilled water at 20 or 40 wt% was added to facilitate the formation of a homogeneous liquid phase. Following the synthesis, NaDESs were characterized based on their appearance, pH, and density, as reported in the Supplementary Materials (Section S1).

### 2.4. Extraction Optimization Using Conventional Solvents

Two extraction methodologies were employed to compare efficiency and optimize the process. Extractions were performed using mixtures of water (H_2_O) and ethanol (EtOH) at ratios of 50:50 and 30:70 (*v*/*v*), with a solid–liquid ratio of 1:10 (i.e., 1 g of lemon waste (L) or orange waste (O) combined with 10 g of solvent). The extractions were conducted as follows:

- Mechanical agitation: Samples were stirred on a roller mixer for 3 h at room temperature (rt) in the dark to prevent photodegradation of bioactive compounds.
- Ultrasound-assisted extraction (UAE): Samples underwent alternating 5 min cycles in an ultrasonic bath followed by 1 min cooling on ice, for a total extraction time of 1 h, to minimize overheating and degradation.

After extraction, all mixtures were centrifuged for 10 min at 1200× *g*, separated from the residual biomass, and filtered through a filter membrane (0.2 μm pores). Samples were stored in the dark at 4 °C. Each extraction was performed in triplicate. Following the production, the extracts were characterized based on their appearance and pH, as reported in the Supplementary Materials (Section S2).

### 2.5. Evaluation of Total Phenolic Content

The total phenolic content (TPC) of extracts was assessed using the Folin–Ciocalteu colorimetric method following the procedure originally reported by Ainsworth et al. [14]. This method relies on the redox reaction between phenolic compounds and the Folin–Ciocalteu reagent. In this process, phenolic compounds act as electron donors, reducing the reagent and forming a blue-colored complex. The color intensity, which correlates with the phenolic content of the sample, can be detected spectrophotometrically.

Briefly, each extract solution (100 μL) was combined with 200 µL of Folin–Ciocalteu reagent and 800 µL of sodium carbonate solution (700 mM). The reaction mixtures were incubated for 2 h at room temperature in the dark. Subsequently, 200 µL of each sample was transferred into a 96-well microplate, and absorbance was measured at 765 nm using a spectrophotometric reader. Distilled water was used as blank. Gallic acid (100–1000 µg/mL) served as the standard compound, and the TPC was expressed as μg of gallic acid equivalent (GAE)/mg citrus extract. Each measurement was performed in triplicate, and the data represent the mean of three independent experiments.

### 2.6. Citrus Waste Extracts Preparation Using NaDESs

O or L was mixed with NaDES at a solid–liquid ratio of 1:10 (*w*/*w*); i.e., 1 g of L/O was added to 10 g of diluted solvents. The suspension was stirred on a roller stirrer at rt, in the dark, for 3 h. After extraction, all mixtures were centrifuged for 10 min at 1200× *g*, separated from the residual biomass, and filtered through a filter membrane (0.2 μm pores). Finally, samples were stored in the dark at 4 °C. Each extraction was performed in triplicate. Sample nomenclature according to solvent used:

1. O_(ChCl: U)_: extract obtained using ChCl: U starting from Orange Matrix;
2. L_(ChCl: U)_: extract obtained using ChCl: U starting from Lemon Matrix;
3. O_(ChCl: CA)_: extract obtained using ChCl: CA starting from Orange Matrix;
4. L_(ChCl: CA)_: extract obtained using ChCl: CA starting from Lemon Matrix;
5. O_(Bet: U)_: extract obtained using Bet: U starting from Orange Matrix;
6. L_(Bet: U)_: extract obtained using Bet: U starting from Lemon Matrix.

Following the extractions, extracts were characterized based on appearance, including crystallization and precipitation phenomena, pH evaluation, as reported in the Supplementary Materials (Section S3), and TPC.

### 2.7. Polyphenol Profile by HPLC-ESI-MS/MS

Liquid chromatography analysis was performed using an Agilent 1290 Infinity II ultra-high-performance liquid chromatography (UHPLC) system, coupled to an Agilent 6495 LC/TQ triple quadrupole mass spectrometer. The system featured an electrospray ionization (ESI) source configured for negative ionization mode and operated in multiple reaction monitoring (MRM) acquisition mode. Analytical separation was performed using Phenyl-Hexyl (1.7 µm, 150 mm × 2.1 mm i.d; Waters, Milford, MA, USA). The mobile phase consisted of solvent A (0.1% formic acid in water) and solvent B (0.1% formic acid in methanol) in gradient elution mode: B 5%, B 35% to 4 min and kept to 8 min, B 40% to 11 min, B 55% to 12 min, B 70% to 13 min and kept to 16 min, B 80% to 17 min and kept to 20 min. The equilibration time was 3 min, and the total run time was 23 min. The flow rate was 0.3 mL/min, the column temperature was maintained at 33 °C, and the injection volume was 2 μL.

The MS/MS parameters were optimized by directly infusing single analytes diluted in the mobile phase. Argon gas was employed as the collision gas, and nitrogen as the nebulizer and heater gas. The nitrogen flow rates were set to 14 L/min for nebulization and 12 L/min for desolvation. The ion source block and sheath gas temperature were set at 220 °C and 350 °C, respectively. Capillary and nozzle voltages were adjusted to 3.00 kV and 2.00 kV, respectively.

Standard solutions of five polyphenols were used to evaluate the polyphenol profiles of each extract. Single stock solutions (1 mg/mL) of rutin, naringin, naringenin, hesperetin, and hesperidin were prepared in ethanol or dimethyl sulfoxide (DMSO) and stored until use at −80 °C.

Isotopically labeled internal standards (ISs) were included. Rutin-d3, hesperetin-13C-d4, and naringenin-d4 were prepared in DMSO at 1 mg/mL and stored until use at −80 °C. Table 2 reports the optimized MS/MS conditions for each analyte and its internal standard, detailing the selected precursor and product ions as well as the corresponding collision energies.

Standard solutions used for method validation were prepared by diluting stock solutions in the mobile phase. Spiked sample solutions (QC) used for matrix-matched calibration curve were obtained by adding diluted stock solution in the range 2–100 ng/mL to the solvent (ChCl: U; ChCl: CA, Bet: U).

The limit of detection (LOD) and limit of quantification (LOQ) were established based on signal-to-noise ratios of 3 and 10, respectively. Matrix effects for each compound were assessed by performing an ANOVA test to compare six-point calibration curves (2, 5, 10, 25, 50, and 100 ng/mL) prepared in mobile phase and in ChCl: U; ChCl: CA, Bet: U, respectively.

Selectivity was assessed by comparing the chromatograms obtained from standards, samples, and spiked sample solutions. ISs were employed for calibration curves at a fixed concentration of 10 ng/mL.

Accuracy was expressed as bias (%) = (STDm-STDs)/STDs, where STDm and STDs represent the mean measured concentration and spiked concentration, respectively. Precision was calculated as the coefficient of variation (CV%).

### 2.8. Human Keratinocytes Cellular Model

The human keratinocyte cell line (HaCaT), spontaneously immortalized and derived from adult epidermis, was used for all in vitro experiments. The cells were kindly provided by Prof. F. Martini (University of Ferrara, Italy) and originally purchased from the American Type Culture Collection (ATCC, Manassas, VA, USA). Cells were maintained in high-glucose DMEM supplemented with 10% fetal bovine serum (FBS), 2.5 mM L-glutamine, and penicillin/streptomycin (100 U/mL/100 µg/mL) at 37 °C in a humidified incubator with 5% CO_2_. The culture medium was refreshed daily before experiments to promote cell growth and viability.

### 2.9. Cell Cytotoxicity and Cell Viability

Cellular cytotoxicity and viability were evaluated using two complementary colorimetric assays, based, respectively, on lactate dehydrogenase (LDH) release and WST-8 [2-(2-methoxy-4-nitrophenyl)-3-(4-nitrophenyl)-5-(2,4-disulfophenyl)-2H-tetrazolium, monosodium salt] reduction. The combination of these methods allowed a comprehensive assessment of both membrane integrity and metabolic activity in HaCaT cells following treatment with the extracts.

The detection of LDH release from cells was evaluated in the culture medium using the Cytotoxicity LDH Assay Kit (Dojindo Molecular Technologies, Kumamoto, Japan). In the reaction, LDH catalyzes the oxidation of lactate to pyruvate, simultaneously reducing NAD^+^ to NADH. The generated NADH then drives a diaphorase-dependent conversion of a tetrazolium salt into a red formazan product. The amount of formazan, quantified spectrophotometrically at 490 nm, is directly proportional to the extent of cell membrane disruption.

For these experiments, HaCaT cells were seeded in 96-well plates (10 × 10^4^ cells/well) and cultured at 37 °C with 5% CO_2_. After 24 h, the cells were treated for 24 h with different dilution of extracts O_(ChCl: U)_, L_(ChCl: U)_, O_(ChCl: CA)_, L_(ChCl: CA)_, O_(Bet: U)_, and L_(Bet: U)_, (dilution range of 1:10–1:1000) and then the culture medium was collected. The change in the absorbance between treated samples and controls was monitored at 37 °C using an Allsheng FlexA-200 Microplate Reader (Allsheng, Hangzhou, China). Each measurement was performed in triplicate, and the data represent the mean of three independent experiments.

Table S4 summarizes the calculated concentrations (µg/mL) of the major identified phenolic compounds for each extract and dilution, allowing direct comparison of dose levels across treatments.

To complement LDH results and estimate metabolic activity, cell viability was measured using the WST-8 assay. In this colorimetric bioassay, the salt WST8 is reduced by cellular dehydrogenases to orange, water-soluble formazan product, whose absorbance at 450 nm directly reflects the number of living cells.

For these experiments, HaCaT cells were seeded and treated under the same experimental conditions as described for the LDH using the extracts O_(ChCl: U)_, L_(ChCl: U)_, O_(Bet: U)_, and L_(Bet: U)_ in the dilution range of 1:100–1:1000. The decrease in absorbance between the samples and the control was monitored at 450 nm using an Allsheng FlexA-200 Microplate Reader at 37 °C. Each measurement was performed in triplicate, and the data represent the mean of three independent experiments.

### 2.10. Antioxidant Activity of the Extracts in HaCaT Cells

The intracellular H_2_O_2_ production was assessed in HaCaT cells through the chemiluminescent (CL) cell-based bioassay previously developed by our group [15]. The working solution of the probe and the pro-oxidant stimuli (menadione or LPS) were prepared in PBS from their respective stock solutions. Before testing, calibration curves were obtained to verify the linear relationship between probe emission and H_2_O_2_ concentration in HaCaT cells exposed to increasing doses of the pro-oxidants, as reported in the Supplementary Materials (Section S5).

Once the method was optimized in HaCaT cells, the antioxidant activities of the extracts were investigated. HaCaT cells (10 × 10^4^ cells/well) were seeded in a 96-well black plate with a transparent bottom. The following day, the cells were treated for 24 h with extracts O_(ChCl: U)_, L_(ChCl: U)_, O_(Bet: U)_, and L_(Bet: U)_ (dilution range: 1:100–1:1000) and hydroxytyrosol (HT) (concentration range: 0.5–25 µM) as positive antioxidant control [16]. Following treatment, the medium was replaced with 50 µL of probe working solution (final concentration of 5 μM), and the plates were incubated for 20 min at 37 °C. Subsequently, oxidative stress was induced by adding menadione (50 µM) or LPS (25 µg/mL) to the wells. CL emission was monitored for 40 min using a Varioskan Flash plate reader (Thermo Fisher Scientific, Waltham, MA, USA). The PBS solution was used as a negative control. Each measurement was performed in triplicate, and the data represent the mean of three independent experiments.

### 2.11. The Scratch Assay in HaCaT Cells

HaCaT cells (80 × 10^4^ cells/well) were seeded into 6-well transparent plates and cultured until reaching approximately 80% confluence. A uniform scratch was then generated through the cell monolayer using a sterile 200 µL pipette tip. Detached cells and debris were removed by gentle washing with PBS, and fresh complete medium was added.

Images of the scratched area were captured immediately after the scratch (time 0) using an Olympus CL40 inverted microscope connected to an electron-multiplying charge-coupled device (EMCCD) camera (ImagEM-X2, Hamamatsu). After the scratch formation, the culture medium was supplemented with the various extracts (O_(ChCl: U)_, L_(ChCl: U)_, O_(Bet: U)_, L_(Bet: U)_) at a dilution of 1:100. The cells were maintained at 37 °C in a humidified atmosphere containing 5% CO_2_ throughout the experiment. The wound area was re-examined and imaged at 24 h and 48 h post-treatment to assess the rate of wound closure. Untreated cells served as the negative control. Images were analyzed using ImageJ (version 1.54p; NIH, Bethesda, MD, USA). The wound zone was outlined, and its area measured (µm^2^) at each time point. For each condition, three independent wound regions were imaged per well, and the average wound area was used for the calculation. Wound closure was quantified using ImageJ software and calculated according to Equation (1).

(1) $${\mathrm{Wound}\;\mathrm{Closure}}_{\;\%}=\frac{Wound\;area\;(t_{0})-Wound\;area\;(t_{x})}{Wound\;area\;(t_{0})\times 100}$$

This formula quantifies the relative decrease in the wound area over time, providing a direct measure of the keratinocytes’ ability to restore the integrity of the cell monolayer under different treatment conditions [17,18]. Each measurement was performed in triplicate, and the data represent the mean of three independent experiments.

### 2.12. Protein Release

HaCaT cells (80 × 10^4^ cells/well) were seeded for 24 h into 6-well transparent plates. Then, cells were pretreated with extracts (O_(ChCl: U)_, L_(ChCl: U)_, O_(Bet: U)_, L_(Bet: U)_) at a dilution of 1:100 for 24 h. After treatment, the supernatants were collected and centrifuged to eliminate cellular debris. The concentrations of IL-1β, IL-10, IL-8, and IL-6 were quantified using commercial ELISA kits (Elabscience Biotechnology Co., Wuhan, China) following the manufacturer’s instructions. All determinations were performed in triplicate, and data represent the mean of three independent experiments.

### 2.13. Immunofluorescence Determination of 53BP1 Foci

HaCaT (25 × 10^4^ cells/well) were seeded onto glass coverslips, pretreated with O_(Bet: U)_ (dilution 1:100) for 24 h. Subsequently, cells were exposed to the genotoxic agent neocarzinostatin (NCS) (0.13 μM) for 2 h to induce DNA damage, followed by a 24 h recovery period in fresh medium. Cells were then fixed in cold 70% ethanol for 10 min, rinsed once with PBS, and blocked for 30 min in 4% BSA prepared in PBS containing 0.1% Tween-20 (PBST). After blocking, samples were incubated for 1 h at room temperature with the primary antibody anti-53BP1 (Novus Biologicals, Littleton, CO, USA) diluted in 1% BSA-PBST buffer. Following three washes in PBST, slides were incubated for 1 h with Alexa Fluor 555-conjugated goat anti-rabbit secondary antibody (Cell Signaling Technology, Danvers, MA, USA) in 1% BSA–PBST. Nuclei were counterstained with Hoechst 33,342 (Thermo Fisher Scientific, Waltham, MA, USA), and slides were mounted using Vectashield mounting medium (Vector Laboratories, Burlingame, CA, USA). Images were acquired with an Olympus CL40 inverted microscope connected to an electron-multiplying charge-coupled device (EMCCD) camera (ImagEM-X2, Hamamatsu). 53BP1 foci detected within the nuclei were quantified and classified as follows: nuclei containing 0 ≤ foci < 5, 5 ≤ foci < 10, 10 ≤ foci < 20, and foci ≥ 20, in increasing order of DNA damage severity [19]. The distribution of cells across these classes was expressed as percentage values for each treatment. Differences among distributions were assessed using the Chi-square test, while the percentage of cells with >20 foci (high-damage class) was analyzed by one-way ANOVA followed by Tukey’s post hoc test. Data are expressed as mean ± SD from three independent experiments, each performed in triplicate.

### 2.14. Statistical Analysis

Data are expressed as mean ± SD (unless otherwise indicated) from at least three independent biological replicates, each performed in triplicate. Statistical analyses were carried out using GraphPad Prism (GraphPad Prism v6.0, GraphPad Software Inc., La Jolla, CA, USA). Data normality was verified using the Shapiro–Wilk test. Differences among multiple groups were assessed by one-way ANOVA or two-way ANOVA, followed by appropriate post hoc tests (Tukey or Dunnett) as indicated in the figure legends. When appropriate, repeated-measures ANOVA was applied to evaluate changes over time. When appropriate, 95% confidence intervals (CI) were calculated to indicate data variability and the precision of mean estimates. IC_50_ values were determined from non-linear regression analysis of dose–response curves using a four-parameter logistic model. A *p*-value < 0.05 was considered statistically significant.

## 3. Results and Discussion

### 3.1. NaDES Preparation and Characterization

Previous research highlighted that citrus waste is a sustainable source rich in bioactive compounds, especially flavonoids and phenolic acids. However, traditional extraction methods from plant matrices often rely on volatile and hazardous organic solvents, which pose significant environmental and safety concerns. To overcome these issues, in our study we exploited NaDES as a safer, eco-friendly alternative.

NaDES formulations were initially screened for stability, pH, and density. Among the tested formulations, ChCl: U, ChCl: CA, and Bet: U demonstrated the most favorable physicochemical properties and were selected for subsequent extraction experiments.

Detailed characterization data, including appearance, crystallization behavior, pH, and density values for all NaDES formulations, are provided in Supplementary Materials (Table S1).

### 3.2. Optimization of Polyphenols Extraction by Using Conventional Solvents

Two distinct extraction methodologies were implemented to identify the more efficient technique and optimize the extraction process. The first method used mechanical agitation, while the second involved the use of UAE. Both extractions were carried out with a conventional solvent blend of H_2_O and EtOH in ratios of 50:50 and 30:70 (*v*/*v*). These solvent mixtures were selected for their ability to effectively dissolve polyphenolic compounds while prioritizing safety over more toxic alternatives, such as methanol and hexane.

All extracts were clear, homogeneous, and non-viscous, with lower pH values in more aqueous (50:50) mixtures and lemon samples due to higher acid content and increased acid dissociation (Table S2). The extraction efficiency of UAE and roller agitation was quantitatively assessed via TPC using the Folin–Ciocalteu assay (Table 3).

Two-way ANOVA (extraction method × extraction condition) revealed no statistically significant differences (*p* > 0.05), indicating that UAE and roller agitation performed comparably across all matrices and solvent compositions. A trend toward higher TPC values was observed with 70% ethanol, consistent with improved solubilization of phenolic compounds under higher ethanol content [20]. Given the comparable performance, lower energy requirements, simpler scale-up, and reduced environmental impact compared to UAE, roller agitation was selected for subsequent NaDES extractions.

### 3.3. Citrus Waste Extracts by Using NaDESs

Based on previous observations, ChCl: U, ChCl: CA, and Bet: U were selected for the extraction experiments. Next, extractions were performed using the roller agitation method, and the extracts were characterized.

All extracts were clear, homogeneous, free of particulates or crystallization, with slightly acidic pH (4.20–5.60); ChCl: CA extracts showed the lowest pH due to citric acid, yet all remained within acceptable ranges for further biological testing (Table S2). Next, the extraction yield was evaluated by determining the TPC of the obtained extracts using the Folin–Ciocalteu assay. All extracts demonstrated a high polyphenol content (Table 4). The extract O_(Bet: U)_ exhibited the highest TPC, with 11.97 ± 0.11 μg GAE/mg extract.

All extracts obtained with NaDESs showed TPC values ranging from 6.30 to 11.97 μg GAE/mg, generally higher than those from EtOH: H_2_O (5.51–7.65 μg GAE/mg). However, one-way ANOVA comparing NaDESs and conventional ethanol–water extracts revealed no significant differences in TPC (*p* > 0.05), although a clear trend toward improved extraction with NaDESs was observed. These results suggest that NaDESs may serve as a sustainable alternative to conventional ethanol–water mixtures without compromising extraction efficiency.

**Figure 1:**
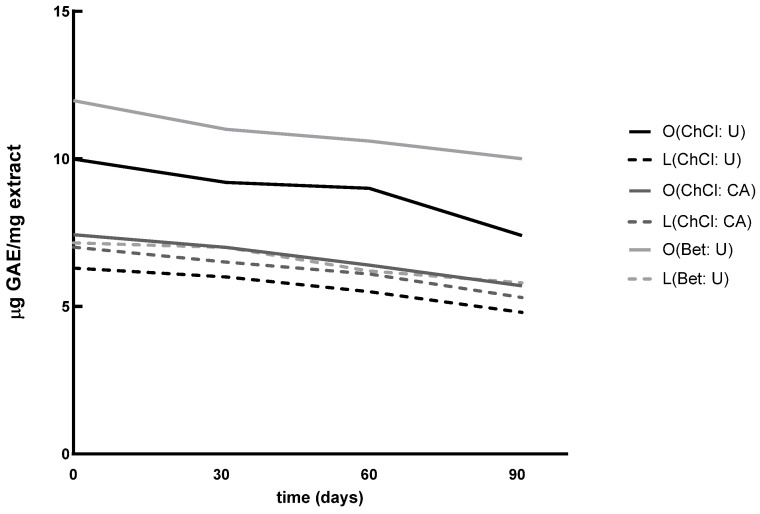
Time-course of Total Phenolic Content (TPC) degradation in NaDES extracts.

Next, TPC was assessed at 30, 60, and 90 days from the initial time point (t_0_). A progressive reduction in phenolic content was observed (Figure 1); however, statistical analysis by repeated-measures ANOVA revealed no significant differences over time (*p* > 0.05). This suggests that, while natural degradation processes occurred, the extracts remained largely stable under the applied storage conditions (protected from light and kept at 4 °C).

### 3.4. Characterization of Citrus Waste Extracts Through HPLC-ESI-MS/MS Analysis

UHPLC-ESI MS/MS determined the polyphenol content in the three formulations. The method was validated using standard samples, QCs, and real samples.

Intra- and inter-day accuracy and precision were within 15% for all analytes at the three tested concentration levels (LOQ, 5× LOQ, and 10× LOQ), confirming the reliability and robustness of the developed method. The comparison of standard solutions, spiked samples, and blank matrices demonstrated good selectivity under MRM acquisition mode.

Due to matrix effects (*p* < 0.05), quantification was performed using matrix-matched calibration curves obtained by plotting the analyte/IS peak area ratio against the analyte concentration, with linear least-squares regression analysis. The determination coefficients (r^2^) of the analytical calibration curves were ≥0.990 for all analytes. LOD and LOQ for all the analytes were 0.1 ng/mL and 1 ng/mL, respectively.

Orange and lemon extracts obtained with the three solvents (ChCl: U, ChCl: CA, Bet: U) were analyzed in triplicate using UHPLC-MS/MS. Before analysis, the extracts were appropriately diluted, and a fixed amount of IS (10 ng/mL) was added to each sample to ensure accurate quantification. Table 5 reports the results obtained.

The data obtained revealed the efficiency of different NaDES formulations in extracting bioactive compounds from citrus waste, both lemon and orange matrices. Each solvent exhibited varying extraction efficiencies, suggesting distinct affinities for specific classes of bioactive molecules.

Rutin was extracted more effectively from lemon waste than orange waste across all NaDES formulations. ChCl: U achieved the highest rutin concentration (23.42 μg/mL), outperforming all other combinations. Naringin was predominantly extracted from orange waste, with the highest yield observed using ChCl: CA (20.14 μg/mL), followed by Bet: U (15.41 μg/mL). In contrast, lemon waste yielded much lower concentrations (0.45–0.95 μg/mL) in all NaDES formulations. Across all samples, naringenin levels were below the limit of quantification (LOQ). This could indicate minimal availability of this aglycone in the analyzed waste materials or challenges in its extraction using these solvents. Hesperetin concentrations were generally low, with the highest levels observed in orange waste (Bet: U, 0.76 μg/mL). Hesperidin showed the highest extraction yield, with Bet: U from orange waste achieving a concentration of 1174.90 μg/mL, significantly surpassing all other combinations. Lemon waste also produced high levels of hesperidin, with yields ranging from 143.10 to 466.00 μg/mL.

The results underline the versatility and efficiency of NaDESs in extracting bioactive compounds from citrus waste. Bet: U emerged as the most promising solvent due to its dual polarity properties, which enable high yields of hesperidin. Further optimization of NaDES formulations could enhance their applications in the nutraceutical and cosmeceutical industries.

### 3.5. Safety Evaluation of Citrus Waste Extracts in HaCaT Cells

The biological activities of the extracts were assessed using the immortalized human keratinocyte cell line HaCaT, a widely recognized in vitro model for studying epidermal homeostasis and pathophysiology [21]. Indeed, due to their stability and high proliferative capacity, HaCaT cells enable reproducible assessment of cellular responses to bioactive compounds.

**Figure 2:**
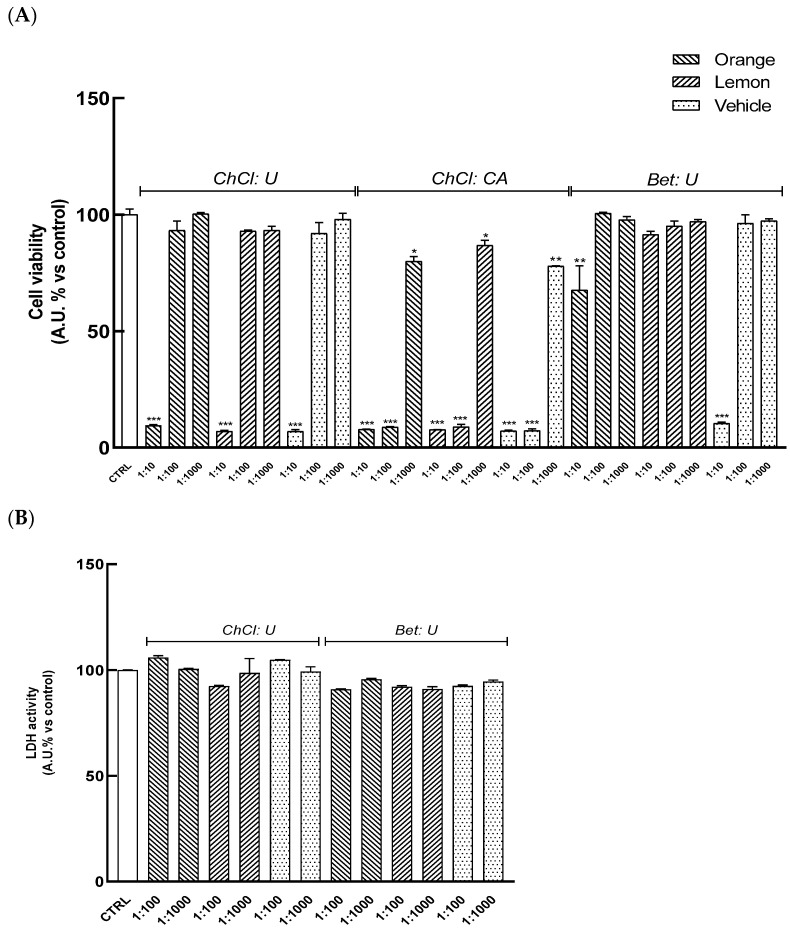
HaCaT cells were pretreated for 24 h with the extracts (dilution range: 1:10–1:1000). (**A**) Cell viability was assessed through the WST-8 assay by detecting formazan formation at 490 nm. (**B**) Cytotoxicity was evaluated by measuring LDH release in the culture medium. Results are expressed as mean ± SD of three independent experiments, each performed in triplicate. Statistical significance was evaluated by one-way ANOVA followed by Dunnett’s test; * *p* < 0.05, ** *p* < 0.01, *** *p* < 0.001 vs. Ctrl (control, untreated cells).

HaCaT cells were pretreated for 24 h with serial dilutions of extracts (range 1:10–1:1000) to evaluate their cytocompatibility. As shown in Figure 2A, treatment with extracts O_(ChCl: CA)_, L_(ChCl: CA)_ results in a significant reduction in cell viability at all tested dilutions. Similarly, the highest concentration (1:10) of other extracts, O_(ChCl: U)_, L_(ChCl: U)_, and O_(Bet: U)_, L_(Bet: U),_ also leads to a significant decrease in formazan dye levels with respect to the control, indicating reduced cellular viability. Conversely, dilutions ranging from 1:100 to 1:1000 of O_(ChCl: U)_, L_(ChCl: U)_, and O_(Bet: U)_, L_(Bet: U)_ did not induce cell toxicity, as also confirmed by the absence of significant changes in LDH release relative to control cells (Figure 2B), indicating these concentrations were safe in HaCat cells.

### 3.6. Evaluation of the Antioxidant Activity of Citrus Waste Extracts in HaCaT Cells

Human skin is consistently exposed to ROS arising from both environmental factors and endogenous processes. Excessive ROS levels damage lipids, proteins, and DNA within cells, impairing the skin’s structure and functions [22]. To assess the antioxidant properties of the extracts, intracellular H_2_O_2_ levels were measured in HaCaT cells through a CL bioassay [11].

Firstly, the correlation between the CL emission and intracellular H_2_O_2_ production was examined by exposing HaCaT cells to increasing concentrations of menadione or lipopolysaccharide (LPS), well-known oxidative stress inducers. Results demonstrated a linear correlation between the CL signal and the concentration of the pro-oxidant stimulus, both for menadione (R^2^ = 0.976) and LPS (R^2^ = 0.989) (Figure S1), confirming the reliability of the method and its alignment with the expected physiological regulation of ROS production.

Next, the assay was applied to investigate the antioxidant capacity of the extracts. HaCaT cells were pretreated for 24 h with different dilutions of the extracts (dilution range: 1:100 to 1:1000), and then exposed to a fixed concentration of menadione (50 μM) or LPS (25 μg/mL). CL emission was recorded for 60 min, and PBS served as the negative control. A concentration-dependent decrease in the CL signal was observed for all extracts, indicating good IC_50_ values, as shown in Table 6.

All extracts effectively reduced ROS levels triggered by both LPS and menadione, as indicated by their IC_50_ values under these conditions. These results suggest that citrus extracts may offer protection against various pro-oxidant challenges, likely due to the combined action of the polyphenolic constituents, which may modulate oxidative stress through multiple mechanisms [23,24].

In parallel, the antioxidant potential of the extracts was also evaluated using the oxygen radical absorbance capacity (ORAC) assay, as described in the Supplementary Materials (Section S6). The ORAC results were consistent with the CL assay, further confirming the antioxidant capacity of the extracts.

### 3.7. Effect of the Extracts on Skin Wound Healing in HaCaT Cells

Tissue repair is a dynamic and finely regulated process involving coordinated cellular responses that restore the integrity and functionality of injured tissues. This process encompasses several stages, including inflammation, cell proliferation, and tissue remodeling, during which various cell lineages, signaling molecules, and growth factors collaborate to restore tissue architecture. Keratinocytes are crucial in this process, as they migrate towards the wound site, undergo proliferation, and produce extracellular matrix components to promote effective tissue regeneration [25,26].

**Figure 3:**
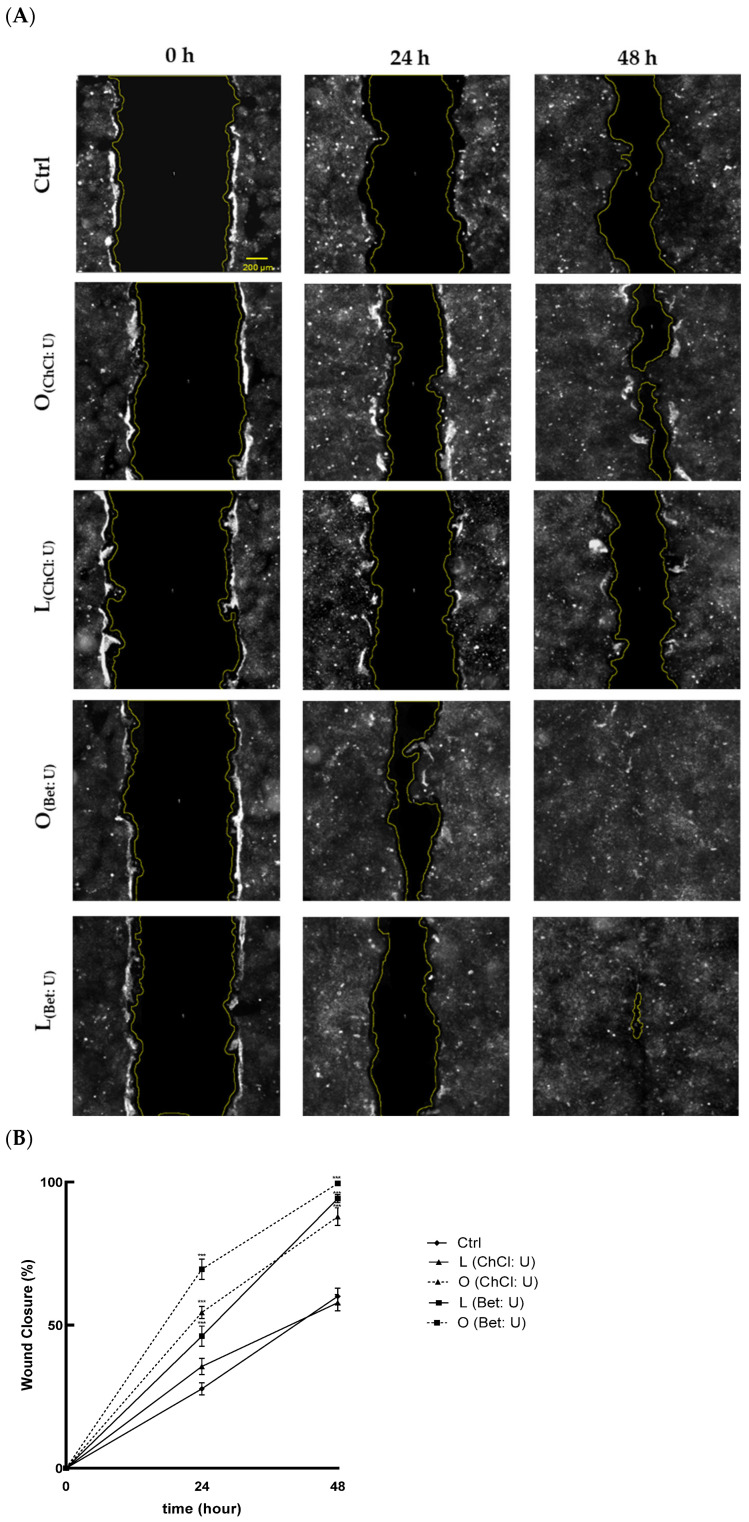
(**A**) Representative figures of scratched confluent layers of HaCaT cells at time 0 h, 24 h, and following 48 h for each treatment using an Olympus CL40 inverted microscope connected to an electron-multiplying charge-coupled device (EMCCD) camera (ImagEM-X2, Hamamatsu, Sunayama-cho, Chuo-ku, Hamamatsu, Japan) under 4× magnification. Scale bar: 200 µm. Ctrl (control, untreated cells); (**B**) Mean ± SD values of Wound Closure (%) of three independent experiments, each performed in triplicate. Significance was determined using one-way ANOVA followed by Tukey’s test; *** *p* < 0.001 vs. Ctrl (control, untreated cells).

To evaluate the effects of the extracts on cell migration and wound healing, a scratch assay was used. As shown in Figure 3A, treatments with O_(ChCl: U)_, O_(Bet: U)_, and L_(Bet: U)_ significantly enhanced wound closure at 24 and 48 h, compared with the control. Notably, treatment with O_(Bet: U)_ achieved near-complete closure, with 72% wound closure after just 24 h and full closure (100%) by 48 h (Figure 3B). In contrast, L_(ChCl: U)_ did not promote significant improvement compared to the control, indicating lower efficacy in promoting keratinocyte migration.

Results indicate that extracts O_(ChCl: U)_, O_(Bet: U)_, and L_(Bet: U)_ significantly promote keratinocyte migration, suggesting enhanced regenerative potential. This wound-healing activity may be primarily linked to their enriched bioactive profiles, especially the high hesperidin content (497.50 μg/mL for O_(ChCl: U)_, 1175.90 μg/mL for O_(Bet: U)_, and 466.00 μg/mL for L_(Bet: U)_). Indeed, several in vitro and in vivo studies have demonstrated that hesperidin, administered either topically or orally, accelerates cutaneous healing under various conditions, including chronic diabetic foot ulcers [23,27]. The antioxidant effect exerted by these extracts may shield keratinocytes from ROS that could otherwise impair the healing process by inducing oxidative stress and compromising cellular integrity [28].

To further elucidate the molecular mechanisms underlying the observed wound-healing effects, RT-qPCR analyses were performed to assess the expression of genes involved in oxidative stress response, inflammation, and tissue remodeling, including TGF-β, NF-κB cofactors (p50, p52, p65), SOD1, and HO-1. As reported in the Supplementary Materials (Section S7, Figure S2), treatment with O_(Bet: U)_ led to the upregulation of SOD1 and HO-1, suggesting activation of the Nrf2/ARE antioxidant pathway, together with a moderate increase in TGF-β, consistent with the promotion of tissue regeneration. Conversely, NF-κB cofactors were only mildly modulated under basal conditions, whereas pretreatment with O_(Bet: U)_ attenuated their overexpression induced by TNF-α, indicating a balanced control of inflammatory signaling. These transcriptional changes support the pro-healing and cytoprotective effects induced by O_(Bet: U)_.

### 3.8. Evaluation of IL-1β, IL-10, IL-8, and IL-6 Cell Release in HaCaT Cells

Wound healing is a carefully orchestrated, multi-phase event involving a balanced interplay between pro-inflammatory and regenerative mediators to achieve effective tissue repair. Cytokines play a central role in regulating keratinocyte and fibroblast proliferation, extracellular matrix remodeling, and immune recruitment of immune cells [29]. To better explore the molecular mechanisms underlying the wound-healing effects of extracts, we measured the release of key interleukins (IL)-1β, -10, -8, and -6, known to modulate inflammatory and regenerative pathways in skin repair, in HaCaT cells treated for 24 h with the extracts using ELISA immunoassays.

**Figure 4:**
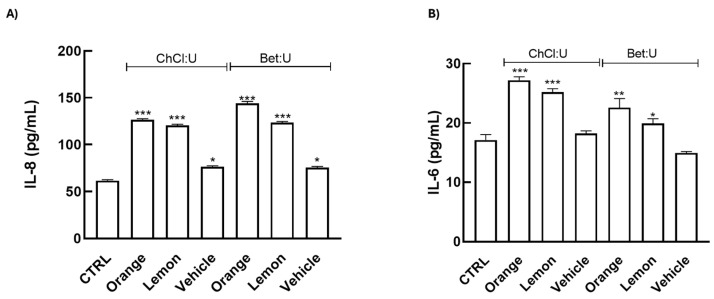
HaCat cells were treated with extracts (1:100) for 24 h. Release of the pro-inflammatory cytokines (**A**) IL-8 (pg/mL) and (**B**) IL-6 (pg/mL) were determined by ELISA assay. Results are expressed as means ± SD of three independent experiments, each performed in triplicate. Significance was determined using one-way ANOVA followed by Dunnett’s test. * *p* < 0.05, ** *p* ˂ 0.01, *** *p* ˂ 0.001 vs. Ctrl (control, untreated cells).

Our data reveal that treatments induce a significant upregulation of IL-6 and IL-8, while IL-1β and IL-10 remained undetectable (Figure 4). IL-6 is a primary early responder following skin injury, rapidly increasing at the wound site and facilitating immune cell recruitment and debris clearance, thereby driving the initial inflammatory phase [30,31]. IL-8, in turn, acts as a potent chemoattractant for neutrophils, enhancing microbial clearance and promoting angiogenesis, critical processes for wound re-epithelialization [32,33,34]. Collectively, these results indicate a beneficial activation of the early inflammatory phase, essential for effective tissue repair.

These findings are noteworthy, as IL-6 and IL-8, traditionally considered pro-inflammatory, are increasingly recognized for their regulatory roles in tissue repair. Both cytokines facilitate keratinocyte migration, fibroblast activation, and neovascularization, essential for the proliferative phase of wound repair [35,36]. Notably, IL-6 also supports macrophage polarization toward a reparative M2 phenotype in the skin, thereby supporting extracellular matrix remodeling and tissue regeneration [30,37]. Consistent with this role, IL-6-deficient animal models display marked delays in wound re-epithelialization, angiogenesis, and collagen deposition [38].

The treatments do not induce IL-1β expression, suggesting that the extracts do not elicit a strong acute inflammatory response, which may otherwise contribute to chronic inflammatory skin conditions [39].

### 3.9. DNA Damage Assessment by 53BP1 Foci Formation in HaCaT Cells

The formation of 53BP1 foci is a well-established marker of DNA double-strand break response and is widely used to monitor cellular genotoxic stress. Maintaining genome integrity during skin regeneration is critical, as keratinocytes and fibroblasts undergo intense proliferation and oxidative stress during the repair process [40]. Persistent accumulation of 53BP1 foci has been linked to cellular senescence and defective DNA-repair mechanisms under chronic oxidative or inflammatory stress [41]. In the skin context, such genomic instability may contribute to delayed tissue regeneration and impaired wound closure [42].

**Figure 5:**
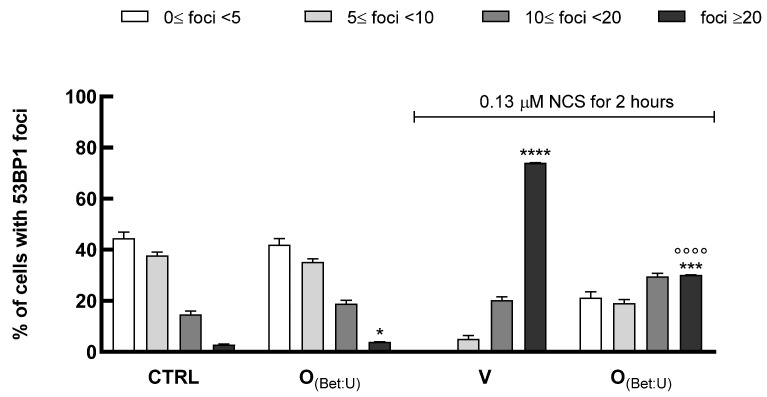
HaCaT cells were pretreated for 24 h with O_(Bet: U)_ (1:100 dilution) and subsequently exposed to neocarzinostatin (NCS) to induce DNA double-strand breaks. Untreated cells served as negative controls, while cells exposed to NCS alone represented the positive control. After 24 h, cells were fixed and stained with an anti-53BP1 antibody and Hoechst for nuclear counterstaining. Cells were categorized according to the number of nuclear 53BP1 foci per cell (0 < foci < 5, 5 < foci < 10, 10 < foci < 20, >20 foci), and the results were expressed as the percentage of nuclei within each class. Histogram bars represent the mean ± SD of three independent experiments, each performed in triplicate. Statistical analysis was performed using the Chi-square test for overall foci distribution and one-way ANOVA followed by Tukey’s post hoc test for the high-damage class (>20 foci per nucleus, * *p* < 0.05, *** *p* < 0.001, **** *p* < 0.0001 vs. Ctrl (control, untreated cells) °°°° *p* < 0.0001 vs. V (cells treated with NCS)).

Therefore, to assess whether the most promising formulation investigated (O_(Bet: U)_) could counteract DNA damage, we performed immunofluorescence analysis of 53BP1 to evaluate its ability to protect keratinocytes from genotoxic stress. In this assay, HaCaT cells were exposed to NCS, in the presence or absence of 24 h pretreatment with O_(Bet: U)_ (dilution 1:100). The number of 53BP1 foci per nucleus was quantified as an indicator of DNA damage levels. As shown in Figure 5, under control conditions, most nuclei exhibited fewer than 10 foci reflecting basal genome stability. In contrast, exposure to NCS markedly shifted the cell population toward nuclei bearing >20 foci, consistent with extensive DNA double-strand breaks. Treatment with O_(Bet: U)_ alone did not alter the distribution compared with control cells, confirming the absence of intrinsic genotoxicity. However, the pretreatment with O_(Bet: U)_ prior to NCS exposure significantly reduced the proportion of cells with >20 foci, suggesting partial protection from NCS-induced DNA damage. Statistical analysis substantiated these findings, with Chi-square test revealing significant differences in the overall distribution of foci among treatments (*p* < 0.001) driven by the NCS-induced damage, whereas control and O_(Bet: U)_ displayed comparable foci distributions (*p* > 0.05). Consistently, the one-way ANOVA applied to the high-damage category (>20 foci per nucleus) confirmed a marked reduction in severely damaged cells after O_(Bet: U)_ pretreatment (*p* < 0.001). Collectively, these results demonstrate that O_(Bet: U)_ markedly attenuates the accumulation of DNA double-strand breaks, plausibly by strengthening cellular resilience to genotoxic stress through the enhancement of antioxidant and cytoprotective pathways, as also indicated by the concomitant upregulation of SOD1 and HO-1, as reported in the Supplementary Materials (Section S7).

## 4. Conclusions

To summarize, this study introduces an innovative and environmentally sustainable approach for valorizing citrus by-products, addressing waste management challenges and meeting the increasing demand for eco-friendly solutions within the cosmetics industry. Using NaDESs, especially ChCl: U and Bet: U, enabled the development of a highly effective extraction method that surpassed traditional ethanol–water systems in isolating important polyphenols such as hesperidin, naringin, and rutin. These findings underscore NaDESs’ ability to extract and concentrate valuable phytochemicals from agricultural waste streams.

According to our data, the extracts showed strong antioxidant activity, significantly improved keratinocyte migration and wound closure, and enhanced cellular protection against genotoxic stress, suggesting their possible use as active ingredients in skin repair and anti-aging products [43]. Notably, the use of biodegradable, cosmetic-grade solvents aligns with green chemistry principles and supports a circular economy. The extraction process, performed at room temperature with a basic roller mixer, offers notable advantages in scalability, energy efficiency, and cost-effectiveness, making industrial applications more scalable.

However, several limitations should be acknowledged. The chemical profile of these extracts remains partially characterized; a more comprehensive analysis using advanced multi-omics approaches (including untargeted metabolomics and proteomics) is needed to elucidate the full spectrum of bioactive molecules and potential synergistic interactions. Furthermore, such analyses are also crucial for verifying the potential presence of contaminants or undesired compounds that may develop during the extraction process and could compromise safety. Additionally, while in vitro tests provide initial insights, they cannot fully reproduce the structural and physiological complexity of human skin. Consequently, ex vivo human skin or advanced models (i.e., reconstructed human epidermis, 3D cultures, organ-on-chip systems) are required to verify the effectiveness and safety in more complex biological systems. Another important consideration concerns formulation stability and delivery. Future studies should investigate the incorporation of NaDES-based extracts into dermatologically relevant systems, such as hydrogels, emulsions, or nanocarriers, to preserve bioactivity, improve skin permeation, and ensure long-term stability and cosmetic compatibility.

In conclusion, this study lays a strong foundation for the sustainable valorization of citrus by-products using environmentally friendly extraction techniques, offering notable environmental and economic advantages. The integration of comprehensive molecular characterization, in vivo validation, and advanced formulation design will be crucial to translating these promising in vitro findings into effective, safe, and market-ready cosmeceutical products. This strategy not only supports circular economy principles but also emphasizes the potential of green chemistry to drive innovation in the cosmeceutical industry.

## Figures and Tables

**Table 1:** Composition, molar ratio, and % Water content of NaDES formulation prepared.

NaDES	HBA	HBD	Abb	Molar Ratio	% Water
1	Choline Chloride	Urea	ChCl: U	1:2	20
2	Choline Chloride	Glucose	ChCl: Glu	2:1	20
3	Choline Chloride	Malic Acid	ChCl: MA	1:1	20
4	Choline Chloride	Citric Acid	ChCl: CA	3:1	20
5	Choline Chloride	Tartaric Acid	ChCl: TA	2:1	20
6	Betaine	Citric Acid	Bet: CA	1:1	40
7	Betaine	Malic Acid	Bet: MA	1:1	30
8	Betaine	Urea	Bet: U	1:1	40

**Table 2:** Optimal MS/MS parameters for each standard and internal standard, including precursor ions, product ions, collision energies and retention times.

Compound	MS/MS	Collision Energy	RT
Rutin	[609.2]→[300]	40	10.8
	[609.2]→[271]	60	
Rutin d3	[612.2]→[303]	40	10.8
Narginin	[579.2]→[271]	40	11.3
	[579.2]→[150.9]	45	
Hesperidin	[609.3]→[301.1]	25	12
	[301.0]→[163.9]	20	
Naringenin	[270.9]→[150.9]	20	14.5
	[270.9]→[118.9]	35	
Naringenin-d4	[275.0]→[150.9]	20	14.4
Hesperetin	[301.0]→[163.9]	25	14.6
	[301.0]→[286.0]	45	
Hesperetin-13C-d4	[305]→[163.9]	25	14.6

**Table 3:** TPC of extracts obtained using ethanol mixtures with UAE and roller agitation.

Solvent	Matrix	TPC μg GAE/mg Extract (±SD)	
		UAE	Roller Stirrer
EtOH: H_2_O (50:50)	Orange	5.54 ± 0.80	5.51 ± 0.40
	Lemon	6.40 ± 1.00	5.60 ± 0.70
EtOH: H_2_O (70:30)	Orange	7.77 ± 0.70	6.94 ± 0.80
	Lemon	8.64 ± 0.90	7.65 ± 0.80

**Table 4:** TPC of extracts obtained with different NaDES formulations; measurements were conducted using the Folin–Ciocalteu.

Sample	TPC (μg GAE/mg Extract) (±SD)
O_(ChCl: U)_	9.99 ± 0.19
L_(ChCl: U)_	6.30 ± 0.13
O_(ChCl: CA)_	7.43 ± 0.12
L_(ChCl: CA)_	7.01 ± 0.14
O_(Bet: U)_	11.97 ± 0.11
L_(Bet: U)_	7.15 ± 0.18

**Table 5:** Amounts of polyphenols extracted in the three tested formulations (means ± standard deviation).

Sample	Rutin μg/mL	Naringin μg/mL	Naringenin μg/mL	Hesperetin μg/mL	Hesperidin μg/mL
O_(ChCl: U)_	2.56 ± 0.34	8.63 ± 1.29	<LOQ	0.33 ± 0.04	497.50 ± 24.87
L_(ChCl: U)_	23.42 ± 2.31	0.45 ± 0.05	<LOQ	0.26 ± 0.02	328.40 ± 14.60
O_(ChCl: CA)_	4.77 ± 0.71	20.14 ± 2.04	<LOQ	0.35 ± 0.07	244.40 ± 12.22
L_(ChCl: CA)_	13.64 ± 1.35	0.95 ± 0.08	<LOQ	0.21 ± 0.02	143.10 ± 7.15
O_(Bet: U)_	4.43 ± 0.68	15.41 ± 1.41	<LOQ	0.76 ± 0.07	1174.90 ± 58.74
L_(Bet: U)_	10.51 ± 1.10	0.51 ± 0.05	<LOQ	0.27 ± 0.02	466.00 ± 22.58

**Table 6:** IC_50_ values evaluated in HaCaT cells using different pro-oxidant stimuli. Hydroxytirosol was used as an antioxidant reference (concentration range of 0.5–25 µM), exhibiting an IC_50_ value of 8.40 µM (corresponding to 1.29 ± 0.19 µg/mL) for menadione, and of 15 µM (corresponding to 2.31 ± 0.38 µg/mL) for LPS. IC_50_ values were obtained by non-linear regression of dose–response curves using GraphPad Prism. Data represent the mean ± SD of three independent experiments, each performed in triplicate.

Sample	IC_50_ (mg/mL) ± SD	
	LPS (25 μg/mL)	Menadione (50 μM)
O_(ChCl: U)_	0.078 ± 0.02	0.219 ± 0.08
L_(ChCl: U)_	0.075 ± 0.03	0.161 ± 0.07
O_(Bet: U)_	0.063 ± 0.03	0.161 ± 0.07
L_(Bet: U)_	0.065 ± 0.02	0.199 ± 0.08

## Data Availability

The raw data supporting the conclusions of this article are not publicly available due to their large size and because some raw files require proprietary software for proper visualization. Data are available from the corresponding author upon request.

## Supplementary Materials

The following supporting information can be downloaded at: https://www.mdpi.com/article/10.3390/nu17233692/s1. Section S1: NaDES Characterization. Section S2: Characterization of Citrus Waste Extracts Obtained Using Conventional Solvents. Section S3: Characterization of Citrus Waste Extracts Obtained Using NaDESs. Section S4: Summary of Phenolic Compound Levels in Each Extract at Different Dilutions. Section S5: Evaluation of Intracellular H2O2 Production in HaCaT Cells. Section S6: Determination of oxygen radical scavenging capacity (ORAC). Section S7: RT-qPCR Analysis of Inflammatory, Antioxidant, and Pro-healing Genes in HaCaT Cells Treated with extract O_(Bet: U)_. References [44,45,46,47,48,49] are cited in the supplementary materials.

## Abbreviations

The following abbreviations are used in this manuscript:

NaDES	Natural Deep Eutectic Solvent
ChCl	Choline Chloride
Bet	Betaine
U	Urea
CA	Citric Acid
MA	Malic Acid
TA	Tartaric Acid
Glu	Glucose
HBA	Hydrogen Bond Acceptor
HBD	Hydrogen Bond Donor
PBS	Phosphate-Buffered Saline
DMEM	Dulbecco’s Modified Eagle Medium
FBS	Fetal Bovine Serum
LDH	Lactate Dehydrogenase
WST-8	Water-Soluble Tetrazolium Salt-8
CL	Chemiluminescence
ROS	Reactive Oxygen Species
H_2_O_2_	Hydrogen Peroxide
LPS	Lipopolysaccharide
HT	Hydroxytyrosol
UHPLC	Ultra High-Performance Liquid Chromatography
ESI	Electrospray Ionization
MS/MS	Tandem Mass Spectrometry
MRM	Multiple Reaction Monitoring
IS	Internal Standard
GAE	Gallic Acid Equivalents
LOQ	Limit of Quantification
LOD	Limit of Detection
SD	Standard Deviation
ANOVA	Analysis of Variance
ELISA	Enzyme-Linked Immunosorbent Assay
IL	Interleukin
NCS	Neocarzinostatin
ORAC	Oxygen Radical Absorbance Capacity
APHH	Anti-Peroxyl Radical Hydrogen-Donating Activity
SOD1	Superoxide Dismutase1
HO-1	Heme Oxygenase-1
TGF-β	Transforming Growth Factor Beta
NF-κB	Nuclear Factor Kappa-Light-Chain-Enhancer of Activated B Cells
Nrf2	Nuclear Factor Erythroid 2–Related Factor 2
53BP1	p53-Binding Protein 1

## References

[1] Panche A.N., Diwan A.D., Chandra S.R. (2016). Flavonoids: An Overview. J. Nutr. Sci..

[2] Tripoli E., La Guardia M., Giammanco S., Majo D.D., Giammanco M. (2007). Citrus Flavonoids: Molecular Structure, Biological Activity and Nutritional Properties: A Review. Food Chem..

[3] Meeting of the Expert Group Fruit and Vegetables Market Observatory—Citrus Fruit. https://agriculture.ec.europa.eu/document/download/d1a1d6c0-bc90-41cd-998a-07c39581e0c7_en?filename=minutes-fv-mo-citrus-2022-11-20_en.pdf.

[4] Kato-Noguchi H., Kato M. (2025). Pesticidal Activity of Citrus Fruits for the Development of Sustainable Fruit-Processing Waste Management and Agricultural Production. Plants.

[5] Marín F.R., Soler-Rivas C., Benavente-García O., Castillo J., Pérez-Alvarez J.A. (2007). By-Products from Different Citrus Processes as a Source of Customized Functional Fibres. Food Chem..

[6] Addi M., Elbouzidi A., Abid M., Tungmunnithum D., Elamrani A., Hano C. (2021). An Overview of Bioactive Flavonoids from Citrus Fruits. Appl. Sci..

[7] Wang L., Wang J., Fang L., Zheng Z., Zhi D., Wang S., Li S., Ho C.-T., Zhao H. (2014). Anticancer Activities of Citrus Peel Polymethoxyflavones Related to Angiogenesis and Others. Biomed. Res. Int..

[8] Goulas V., Manganaris G.A. (2012). Exploring the Phytochemical Content and the Antioxidant Potential of Citrus Fruits Grown in Cyprus. Food Chem..

[9] Silla A., Punzo A., Caliceti C., Barbalace M.C., Hrelia S., Malaguti M. (2025). The Role of Antioxidant Compounds from Citrus Waste in Modulating Neuroinflammation: A Sustainable Solution. Antioxidants.

[10] Silla A., Punzo A., Bonvicini F., Perillo M., Malaguti M., Lorenzini A., Foltran I., Mercatante D., Mandrioli M., Rodriguez-Estrada M.T. (2025). Anti-Inflammatory, Antioxidant and Antibacterial Properties of Tomato Skin and Pomegranate Peel Extracts: A Sustainable Approach for Oral Health Care. Antioxidants.

[11] Punzo A., Porru E., Silla A., Simoni P., Galletti P., Roda A., Tagliavini E., Samorì C., Caliceti C. (2021). Grape Pomace for Topical Application: Green NaDES Sustainable Extraction, Skin Permeation Studies, Antioxidant and Anti-Inflammatory Activities Characterization in 3D Human Keratinocytes. Biomolecules.

[12] Schafer M., Werner S. (2008). Oxidative Stress in Normal and Impaired Wound Repair. Pharmacol. Res..

[13] Dai Y., Witkamp G.-J., Verpoorte R., Choi Y.H. (2015). Tailoring Properties of Natural Deep Eutectic Solvents with Water to Facilitate Their Applications. Food Chem..

[14] Ainsworth E.A., Gillespie K.M. (2007). Estimation of Total Phenolic Content and Other Oxidation Substrates in Plant Tissues Using Folin–Ciocalteu Reagent. Nat. Protoc..

[15] Calabria D., Guardigli M., Mirasoli M., Punzo A., Porru E., Zangheri M., Simoni P., Pagnotta E., Ugolini L., Lazzeri L. (2020). Selective Chemiluminescent TURN-ON Quantitative Bioassay and Imaging of Intracellular Hydrogen Peroxide in Human Living Cells. Anal. Biochem..

[16] Bertelli M., Kiani A.K., Paolacci S., Manara E., Kurti D., Dhuli K., Bushati V., Miertus J., Pangallo D., Baglivo M. (2020). Hydroxytyrosol: A Natural Compound with Promising Pharmacological Activities. J. Biotechnol..

[17] Vidal A., Mendieta Zerón H., Giacaman I., Camarillo Romero M.d.S., López S.P., Meza Trillo L.E., Pérez Pérez D.A., Concha M., Torres-Gallegos C., Orellana S.L. (2015). A Simple Mathematical Model for Wound Closure Evaluation. J. Am. Coll. Clin. Wound Spec..

[18] Balko S., Kerr E., Buchel E., Logsetty S., Raouf A. (2023). A Robust and Standardized Approach to Quantify Wound Closure Using the Scratch Assay. Methods Protoc..

[19] Punzo A., Perillo M., Silla A., Malaguti M., Hrelia S., Barardo D., Caliceti C., Lorenzini A. (2024). Promising Effects of Novel Supplement Formulas in Preventing Skin Aging in 3D Human Keratinocytes. Nutrients.

[20] Ghitescu R.-E., Volf I., Carausu C., Bühlmann A.-M., Gilca I.A., Popa V.I. (2015). Optimization of Ultrasound-Assisted Extraction of Polyphenols from Spruce Wood Bark. Ultrason. Sonochem.

[21] Colombo I., Sangiovanni E., Maggio R., Mattozzi C., Zava S., Corbett Y., Fumagalli M., Carlino C., Corsetto P.A., Scaccabarozzi D. (2017). HaCaT Cells as a Reliable In Vitro Differentiation Model to Dissect the Inflammatory/Repair Response of Human Keratinocytes. Mediat. Inflamm..

[22] Tu Y., Quan T. (2016). Oxidative Stress and Human Skin Connective Tissue Aging. Cosmetics.

[23] Man M.-Q., Yang B., Elias P.M. (2019). Benefits of Hesperidin for Cutaneous Functions. Evid.-Based Complement. Altern. Med..

[24] Martinez R.M., Pinho-Ribeiro F.A., Steffen V.S., Caviglione C.V., Vignoli J.A., Barbosa D.S., Baracat M.M., Georgetti S.R., Verri W.A., Casagrande R. (2015). Naringenin Inhibits UVB Irradiation-Induced Inflammation and Oxidative Stress in the Skin of Hairless Mice. J. Nat. Prod..

[25] Piipponen M., Li D., Landén N.X. (2020). The Immune Functions of Keratinocytes in Skin Wound Healing. Int. J. Mol. Sci..

[26] Werner S., Krieg T., Smola H. (2007). Keratinocyte–Fibroblast Interactions in Wound Healing. J. Investig. Dermatol..

[27] Wang L., He T., Fu A., Mao Z., Yi L., Tang S., Yang J. (2018). Hesperidin Enhances Angiogenesis via Modulating Expression of Growth and Inflammatory Factor in Diabetic Foot Ulcer in Rats. Eur. J. Inflamm..

[28] Bito T., Nishigori C. (2012). Impact of Reactive Oxygen Species on Keratinocyte Signaling Pathways. J. Dermatol. Sci..

[29] Barrientos S., Stojadinovic O., Golinko M.S., Brem H., Tomic-Canic M. (2008). Perspective Article: Growth Factors and Cytokines in Wound Healing. Wound Repair. Regen..

[30] Johnson B.Z., Stevenson A.W., Prêle C.M., Fear M.W., Wood F.M. (2020). The Role of IL-6 in Skin Fibrosis and Cutaneous Wound Healing. Biomedicines.

[31] Mahmoud N.N., Hamad K., Al Shibitini A., Juma S., Sharifi S., Gould L., Mahmoudi M. (2024). Investigating Inflammatory Markers in Wound Healing: Understanding Implications and Identifying Artifacts. ACS Pharmacol. Transl. Sci..

[32] Gillitzer R., Goebeler M. (2001). Chemokines in Cutaneous Wound Healing. J. Leukoc. Biol..

[33] Rennekampff H.-O., Hansbrough J.F., Kiessig V., Doré C., Sticherling M., Schröder J.-M. (2000). Bioactive Interleukin-8 Is Expressed in Wounds and Enhances Wound Healing. J. Surg. Res..

[34] Wilgus T.A., Roy S., McDaniel J.C. (2013). Neutrophils and Wound Repair: Positive Actions and Negative Reactions. Adv. Wound Care.

[35] Kondo T., Ishida Y. (2010). Molecular Pathology of Wound Healing. Forensic Sci. Int..

[36] Eming S.A., Krieg T., Davidson J.M. (2007). Inflammation in Wound Repair: Molecular and Cellular Mechanisms. J. Investig. Dermatol..

[37] Kim S.Y., Nair M.G. (2019). Macrophages in Wound Healing: Activation and Plasticity. Immunol. Cell Biol..

[38] Lin Z.-Q., Kondo T., Ishida Y., Takayasu T., Mukaida N. (2003). Essential Involvement of IL-6 in the Skin Wound-Healing Process as Evidenced by Delayed Wound Healing in IL-6-Deficient Mice. J. Leukoc. Biol..

[39] Cai Y., Xue F., Quan C., Qu M., Liu N., Zhang Y., Fleming C., Hu X., Zhang H., Weichselbaum R. (2019). A Critical Role of the IL-1β–IL-1R Signaling Pathway in Skin Inflammation and Psoriasis Pathogenesis. J. Investig. Dermatol..

[40] Guo S., DiPietro L.A. (2010). Factors Affecting Wound Healing. J. Dent. Res..

[41] Oda T., Gotoh N., Kasamatsu T., Handa H., Saitoh T., Sasaki N. (2023). DNA Damage-induced Cellular Senescence Is Regulated by 53BP1 Accumulation in the Nuclear Foci and Phase Separation. Cell Prolif..

[42] Wilkinson H.N., Hardman M.J. (2020). Senescence in Wound Repair: Emerging Strategies to Target Chronic Healing Wounds. Front. Cell Dev. Biol..

[43] Novotná R., Škařupová D., Hanyk J., Ulrichová J., Křen V., Bojarová P., Brodsky K., Vostálová J., Franková J. (2023). Hesperidin, Hesperetin, Rutinose, and Rhamnose Act as Skin Anti-Aging Agents. Molecules.

[44] Cannavacciuolo C., Pagliari S., Frigerio J., Giustra C.M., Labra M., Campone L. (2022). Natural Deep Eutectic Solvents (NADESs) Combined with Sustainable Extraction Techniques: A Review of the Green Chemistry Approach in Food Analysis. Foods.

[45] Cao G., Alessio H.M., Cutler R.G. (1993). Oxygen-Radical Absorbance Capacity Assay for Antioxidants. Free Radic Biol. Med..

[46] Ambrozova N., Ulrichova J., Galandakova A. (2017). Models for the Study of Skin Wound Healing. The Role of Nrf2 and NF-ΚB. Biomed. Pap..

[47] Amento E.P., Beck L.S. (2007). TGF-β and Wound Healing. Ciba Found Symp.

[48] Süntar I., Çetinkaya S., Panieri E., Saha S., Buttari B., Profumo E., Saso L. (2021). Regulatory Role of Nrf2 Signaling Pathway in Wound Healing Process. Molecules.

[49] Iuchi Y., Roy D., Okada F., Kibe N., Tsunoda S., Suzuki S., Takahashi M., Yokoyama H., Yoshitake J., Kondo S. (2010). Spontaneous Skin Damage and Delayed Wound Healing in SOD1-Deficient Mice. Mol. Cell Biochem..

