# Supplementary material for: Upcycling of Citrus Waste by Natural Deep Eutectic Solvents: Green Extraction of Bioactive Compounds with Antioxidant and Regenerative Properties on Human Keratinocytes
Source: Nutrients. 2025 Nov 25;17(23):3692. doi: 10.3390/nu17233692 (PMC12693630; doi:10.3390/nu17233692)
Supplement: Supplementary file 1 [file nutrients-17-03692-s001.zip › nutrients-3955008-supplementary.pdf]

Supplementary Materials

S1. NaDES Characterization

S1.1 Materials and Methods

Following the synthesis, NaDES were characterized based on their appearance, including crystallization and precipitation phenomena. Next, the pH measurement of NADES solutions was conducted at room temperature (rt) using a Beckman PHI 40 PH Meter. Then, the density of NADESs ($\varrho_{(NaDES)}$) was measured gravimetrically using a 5 mL measuring cylinder at room temperature. The mass ($M_{(NaDES)}$) and volume ($V_{(NaDES)}$) were carefully measured for density calculation by Eq. (S1).

$$\rho_{(NaDES)} = \frac{M(NaDES)}{V(NaDES)} \quad \text{(S1)}$$

S1.2 Results and Discussion

The NaDES formulations that remained stable throughout the observation period were ChCl: U, ChCl: MA, ChCl: CA, ChCl: TA, and Bet: U, all of which retained a clear, residue-free appearance. In contrast, the formulations ChCl: Glu, Bet: MA, and Bet: TA underwent crystallization phenomena. This difference in stability is likely due to the molecular interactions within each NaDES; the stable formulations may have a more balanced eutectic composition, while those exhibiting crystallization phenomena could undergo phase separation or supersaturation under the observed conditions. Of these data, NaDES ChCl: Glu, Bet: MA, and Bet: TA were excluded from further analysis.

Subsequently, the pH and density were measured, and Table S1 presents the values obtained for the NaDES examined at rt.

Table S1. pH and Density Values of Stable NaDES Formulations

NaDES	Sample name	pH Values (±SD)	Density (g/mL) (±SD)
1	ChCl: U	4.50 ±0.08	1.16 ±0.03
3	ChCl: MA	5.00 ±0.07	1.32 ±0.05
4	ChCl: CA	4.20 ±0.10	1.19 ±0.06
5	ChCl: TA	5.00 ±0.12	1.34 ±0.03
8	Bet: U	5.10 ±0.10	1.17 ±0.04

The pH of the solvents is crucial, as it affects chemical reactivity, stability, and eco-compatibility. All NaDES tested fell within a slightly acidic range (from 4.2 to 5.1), meeting biocompatibility requirements and minimizing environmental impact [1], thus supporting their suitability for further biological applications.

Density is a key physical property for solvents, directly impacting the efficiency of mass transfer and solubilization in extraction processes [2]. NaDES formulations exhibit density values ranging from 1.16 to 1.34 g/mL, as shown in Table S1. Among NaDES, ChCl: MA (1.32 ±0.05 g/mL) and ChCl: TA (1.34 ±0.04 g/mL) exhibited the highest densities, likely due to malic and tartaric acids' molecular weights and multiple hydrogen bonding sites, which facilitate dense, tightly packed eutectic structures. Conversely, ChCl: U (1.16 ±0.03 g/mL) and Bet: U (1.17 ±0.04 g/mL) displayed lower densities, suggesting more open molecular structures, which may enhance solubilization and diffusion. ChCl: CA (1.19 ±0.06 g/mL) presents a moderate density, balancing structural compactness and solvent accessibility, thus serving as a versatile candidate. Based on these observations, ChCl: U, ChCl: CA, and Bet: U were selected for subsequent extraction experiments.

S2. Characterization of Citrus Waste Extracts Obtained Using Conventional Solvents

S2.1 Materials and Methods

Following the extraction, all extracts were characterized based on their appearance, including crystallization and precipitation phenomena. Next, the pH measurement of the solutions was conducted at rt using a Beckman PHI 40 PH Meter.

S2.2 Results and Discussion

The extracts were characterized based on appearance, precipitation behavior, and pH.

- *Appearance*: all extracts appear clear, free from particulate matter, homogeneous, and non-viscous.
- *pH evaluation*: as shown in Table S2, the pH of the extracts was similar between the roller agitation and ultrasound-assisted extraction (UAE) methods. Extracts obtained with the 50:50 ethanol–water mixture were consistently more acidic than those prepared with the 70:30 mixture. This is attributed to the higher water content, which promotes the dissociation of organic acids and proton release, thereby lowering the pH, whereas the less aqueous 70% ethanol reduces acid ionization. Additionally, extracts from lemon samples were generally more acidic than those from orange samples, likely due to their higher citric acid content.

Table S2. pH values of extracts obtained with ethanol-water mixture; measurements were conducted using a Beckman pH meter.

Solvent	Matrix	pH Values (±SD)	
		UAE	Roller Stirrer
EtOH: H_2O (50:50)	Orange	5.10 ±0.08	4.80 ±0.09
	Lemon	4.40 ±0.09	4.54 ±0.05
EtOH: H_2O (70:30)	Orange	5.66 ±0.10	5.64 ±0.06
	Lemon	5.23 ±0.12	5.77 ±0.03

S3. Characterization of Citrus Waste Extracts Obtained Using NaDESs

S3.1 Materials and Methods

Following the extraction, all extracts were characterized based on their appearance, including crystallization and precipitation phenomena. Next, the pH measurement of solutions was conducted at rt using a Beckman PHI 40 PH Meter.

S3.2 Results and Discussion

The extracts were characterized based on appearance, precipitation behavior, and pH.

- *Appearance*: all extracts appear clear, free from particulate matter, homogeneous, and with no signs of precipitation or crystallization.
- *pH evaluation:* all extracts presented pH values within a slightly acidic range from 4.20 to 5.60 (Table S3). Notably, extracts obtained with ChCl: CA exhibited the lowest pH among the formulations, likely due to the presence of citric acid, known for its acidic properties. However, extracts remained within an acceptable pH threshold, suggesting their compatibility for further biological testing.

Table S3. pH values of extracts obtained with different NaDES formulations; measurements were conducted using a Beckman pH meter.

Sample	pH Values (±SD)
$O_{(ChCl: U)}$	5.60 ±0.09

$L_{(ChCl: U)}$	5.10 ±0.10
$O_{(ChCl: CA)}$	4.40 ±0.10
$L_{(ChCl: CA)}$	4.20 ±0.09
$O_{(Bet: U)}$	5.40 ±0.12
$L_{(Bet: U)}$	5.10 ±0.10

S4. Summary of Phenolic Compound Levels in Each Extract at Different Dilutions

Table S4 summarizes the calculated concentrations (µg/mL) of the major identified phenolic compounds for each extract and dilution, allowing direct comparison of dose levels across treatments.

Table S4. Calculated concentrations (µg/mL) of major identified phenolic compounds in each extract and dilution.

**Sample**	**Phenolic compound**	Stock solution (µg/mL)	Dilution 1:10 (µg/mL)	Dilution 1:100 (µg/mL)	Dilution 1:500 (µg/mL)	Dilution 1:1000 (µg/mL)
$O_{(ChCl: U)}$	Rutin	2.56	0.26	0.03	0.01	2.56 x10^{-3}
	Naringin	8.63	0.86	0.09	0.02	0.01
	Naringenin	<LOQ	<LOQ	<LOQ	<LOQ	<LOQ
	Hesperetin	0.33	0.03	3.30 x10^{-3}	6.60 x10^{-4}	3.30 x10^{-4}
	Hesperidin	497.50	49.75	4.98	1.00	0.50
$L_{(ChCl: U)}$	Rutin	23.42	2.34	0.23	0.05	0.02
	Naringin	0.45	0.05	4.50 x10^{-3}	9.00 x10^{-4}	4.50 x10^{-4}
	Naringenin	<LOQ	<LOQ	<LOQ	<LOQ	<LOQ
	Hesperetin	0.26	0.03	2.60 x10^{-3}	5.20 x10^{-4}	2.60 x10^{-4}
	Hesperidin	328.40	32.84	3.28	0.66	0.33
$O_{(ChCl: CA)}$	Rutin	4.77	0.48	0.05	9.54 x10^{-3}	0.01
	Naringin	20.14	2.01	0.20	0.04	0.02
	Naringenin	<LOQ	<LOQ	<LOQ	<LOQ	<LOQ
	Hesperetin	0.35	0.04	3.50 x10^{-3}	7.00 x10^{-4}	3.50 x10^{-4}
	Hesperidin	244.40	24.44	2.44	0.50	0.24
$L_{(ChCl: CA)}$	Rutin	13.64	1.36	0.14	0.03	0.01
	Naringin	0.95	0.10	9.50 x10^{-3}	1.90 x10^{-3}	9.50 x10^{-4}
	Naringenin	<LOQ	<LOQ	<LOQ	<LOQ	<LOQ
	Hesperetin	0.21	0.02	2.10 x10^{-3}	4.20 x10^{-4}	2.10 x10^{-4}
	Hesperidin	143.10	14.31	1.43	0.29	0.14
$O_{(Bet: U)}$	Rutin	4.43	0.44	0.04	0.01	4.43 x10^{-3}
	Naringin	15.41	1.54	0.15	0.03	0.02
	Naringenin	<LOQ	<LOQ	<LOQ	<LOQ	<LOQ
	Hesperetin	0.76	0.08	7.60 x10^{-3}	1.52 x10^{-3}	7.60 x10^{-4}
	Hesperidin	1174.90	117.49	11.75	2.35	1.18
$L_{(Bet:U)}$	Rutin	10.51	1.05	0.11	0.02	0.01
	Naringin	0.51	0.05	5.10 x10^{-3}	1.02 x10^{-3}	5.10 x10^{-4}
	Naringenin	<LOQ	<LOQ	<LOQ	<LOQ	<LOQ
	Hesperetin	0.27	0.03	2.70 x10^{-3}	5.40 x10^{-4}	2.70 x10^{-4}
	Hesperidin	466.00	46.60	4.66	0.93	0.47

S5. Evaluation of Intracellular H_2O_2 Production in HaCaT Cells

S5.1 Materials and Methods

The intracellular H_2O_2 production was estimated in HaCaT cells through the chemiluminescent (CL) cell-based bioassay previously described by our group [3]. The working solutions of the CL probe and the pro-oxidant agents menadione and LPS were prepared by diluting the respective stock solutions with PBS. Firstly, the correlation between the CL emission and the production of H_2O_2 in HaCaT cells, stimulated with different concentrations of menadione or LPS used as oxidative stress inducers, was assessed.

For this set of experiments, HaCaT cells (10 × 10^4 cells/well) were seeded in a 96-well black plate with a transparent bottom. After 24 h, cells were incubated with CL probe (final concentration: 5 µM) for 20 min at 37 °C and then injured with different concentrations of menadione (final concentration range: 2.50—50 µM) or LPS (final concentration range: 3.12—25 µg/mL). CL emission was monitored for 40 min using a Varioskan Flash plate reader (Thermo Fisher Scientific, Waltham, MA). The PBS solution was used as a negative control. The kinetic profiles of CL emission were recorded, and a calibration curve was constructed by plotting the CL signal against the actual concentration of the pro-oxidant agent. The experimental data were fitted to a straight line using the least squares method.

S5.2 Results and Discussion

As illustrated in Figure S1 A, the kinetic profiles of CL emission were analyzed, and the calibration curve, obtained by plotting the CL signal versus the actual concentration of the pro-oxidant, demonstrated a strong positive linear correlation, both with menadione (R^2 = 0.976) and LPS (R^2 = 0.989), reflecting physiological mechanisms of intracellular H_2O_2 production (Fig. S1 B, C).

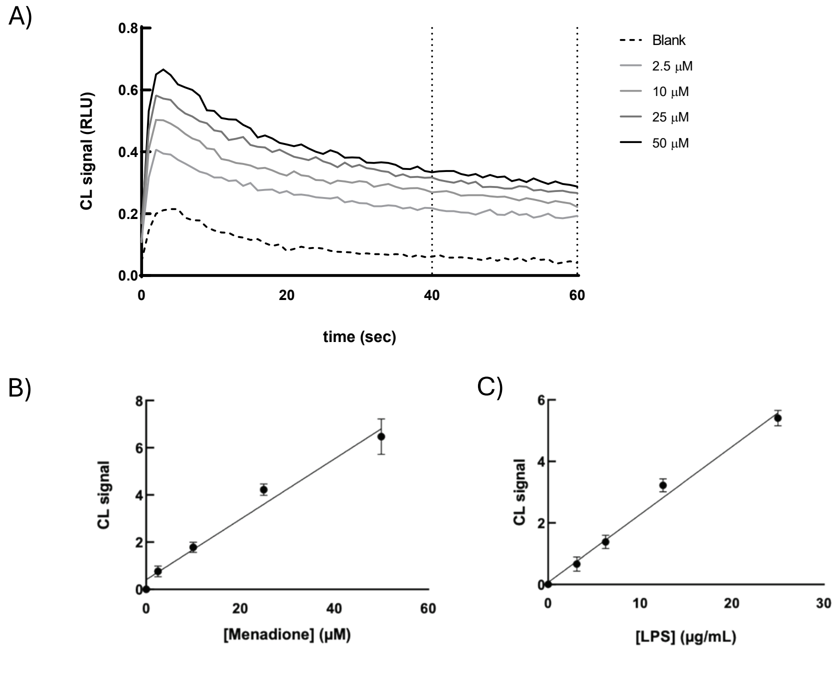

Figure S1. (**A**) CL kinetic profiles were obtained for HaCaT cells in the presence of different concentrations of menadione. (**B**) Dose-response curve showing the correlation between the CL signal and the concentration of menadione. (**C**) Dose-

response curve showing the correlation between the CL signal and the concentration of LPS. Each point represents the mean ± SD of three independent experiments, each performed in triplicate.

S6. Determination of oxygen radical scavenging capacity (ORAC)

S6.1 Materials and Methods.

The antioxidant activity of the extracts was evaluated using the oxygen radical absorbance capacity (ORAC) assay [4], with fluorescein as the fluorescent probe. Briefly, 50 µL of blank, Trolox as standard solutions (10–50 µM), and extract dilutions (1:10–1:1000) were transferred into triplicate wells of a black 96-well microplate. After incubation at 37 °C for 10 min, 50 µL of fluorescein solution (78 nM) was added to each well and incubated for an additional time of 20 min at 37 °C. The reaction was initiated by adding 25 µL of freshly prepared 2,2′-Azobis(2-amidinopropane) dihydrochloride (AAPH) (221 mM) to each well, and the fluorescence decay (excitation 485 nm; emission 535 nm) was monitored every 5 min for 1 h using a Varioskan Flash plate reader (Thermo Fisher Scientific, Waltham, MA, USA).
Final ORAC values were calculated from the calibration curve obtained by plotting the area under the fluorescence decay curve (AUC), after subtraction of the blank, versus Trolox concentration. Linear regression was used to fit the standard data. The antioxidant capacity of the extracts was determined by interpolating their AUC values on the Trolox calibration curve and correcting for the respective dilution factors. Results were expressed as micromoles of Trolox equivalents per milligram of extract (µmol TE/mg extract) and are reported as the mean ± SD of three independent experiments.

S6.2 Results and Discussion

The antioxidant capacity of the extracts was evaluated using the ORAC assay, and the results were expressed as micromoles of Trolox equivalents per milligram of extract (µmol TE/mg extract) (Table S5).

Table S5. Oxygen Radical Absorbance Capacity (ORAC) values of extracts. Results are expressed as micromoles of Trolox equivalents per milligram of extract (µmol TE/mg extract) at a 1:100 dilution. Data represent the mean ±SD of three independent experiments, each performed in triplicate.

NaDES	Matrix	µmol TE/mg (±SD)
ChCl: U	O	57.60 ±4.12
	L	25.06 ±3.1055
Bet: U	O	75.32 ±3.17
	L	55.72 ±3.37

All samples showed appreciable antioxidant potential, with distinct differences depending on both the NaDES composition and the citrus matrices. The highest ORAC value was observed for the $O_{(Bet: U)}$ extract (75.32 µmol TE/mg), followed by $O_{(ChCl: U)}$ (57.60 µmol TE/mg) and $L_{(Bet: U)}$ (55.72 µmol TE/mg). The $L_{(ChCl: U)}$ extract showed the lowest antioxidant capacity (25.06 µmol TE/mg). These results indicate that orange-derived extracts consistently exhibit higher radical scavenging ability compared to their lemon counterparts, suggesting a greater extraction or preservation of antioxidant molecules such as flavanones and phenolic acids.
Overall, the observed ORAC values confirm that the use of NaDES systems enables the recovery of highly active antioxidant fractions from citrus by-products. These data support the potential of these green solvents as sustainable alternatives to conventional organic solvents for the extraction of antioxidant phytochemicals.

S7. RT-qPCR Analysis of Inflammatory, Antioxidant, and Pro-healing Genes in HaCaT Cells Treated with extract $O_{(Bet: U)}$

S7.1. Materials and Methods

S7.1.1. RNA extraction

HaCaT cells (1 × 10^6 cells/well) were seeded in six-well plates and cultured at 37 °C with 5% CO_2. After 24 h, the cells were pretreated for 24 h with $O_{(Bet: U)}$ (dilution 1:100). Subsequently, the culture medium was replaced, and TNF*α* (10 ng/ml) was added for 16 h to induce inflammation. Total RNA was extracted using the RNeasy Mini Kit following the manufacturer's protocol. RNA quantity and purity were determined spectrophotometrically with a NanoDrop One instrument (Thermo Fisher Scientific, Waltham, MA, USA).

S7.1.2. Real-Time PCR

For gene expression analysis, 25 ng of total RNA from each sample were reverse-transcribed and amplified using the Power SYBR™ Green RNA-to-CT™ 1-Step Kit, according to the manufacturer's protocol, in a final reaction volume of 20 μL. Amplification reactions were performed on a QuantStudio 1 Real-Time PCR System (Thermo Fisher Scientific, Waltham, MA, USA) under the following cycling conditions: reverse transcription at 45 °C for 10 min, initial denaturation at 95 °C for 2 min, followed by 40 cycles of denaturation (95 °C for 5 s) and annealing/extension (60 °C for 20 s). Data were analyzed using QuantStudio 1 System Software. Primer concentration was 50 μM.

The human primer sequences were as follows:

- Ribosomal protein L13A (RPL13A): forward 5′-CACCCTGGAGGAGAAGAGGA-3′, reverse 5′-CCGTAGCCTCATGAGCTGTT-3′;
- Superoxide dismutase 1 (SOD1): forward 5′-AGGCATGTTGGAGACTTGGG-3′, reverse 5′-TGCTTTTTCATGGACCACCAG-3′;
- p50: forward 5'-AAT GGG CTA CAC CGA AGC AA-3′, reverse 5'-AGC TCG TCT ATT TGC TGC CT-3';
- p52: forward 5'-CCG TTG TAC AAA GAT ACG CGG-3', reverse 5'-CAT CCA GAC CTG GGT TGT AGC-3';
- p65: forward 5'-TGG GGA CTA CGA CCT GAA TG-3', reverse 5'-GGG GGC ACG ATT GTC AAA GA-3';
- Heme oxygenase-1 (HO-1): forward 5′-CAACAAAGTGCAAGATTCTG-3′, reverse 5′-GATTCACATGGCATAAAG-3′;
- Transforming Growth Factor-Beta (TGF-β) forward 5'-CAACTATTGCTTCAGCTCCACG-3′, reverse 5'-AAGTTGGCATGGTAGCCCTT-3'.

Relative expression levels were calculated using the $2^{-}\Delta\Delta Ct$ method, with RPL13A serving as the internal reference gene. Each measurement was performed in triplicate, and the data represent the mean of three independent experiments, each performed in triplicate. Statistical analysis was performed by one-way ANOVA followed by Dunnett's post hoc test, with p <0.05 considered statistically significant.

S7.2 Results and discussion

Upon treatment with $O_{(Bet:U)}$, an up-regulation of p65 and p50 expressions was observed relative to untreated controls (p <0.001 and p <0.01, respectively), whereas p52 expression remained unaltered (p >0.05) (Figure S2). These findings suggest a selective and limited activation of the canonical NF-κB pathway, with no detectable involvement of the non-canonical arm. Moreover, TNF*α* stimulation induced a strong up-regulation of p65, p50, and especially p52 (p <0.0001), confirming robust NF-κB activation under inflammatory stress. Pretreatment with $O_{(Bet:U)}$ significantly counteracted this response (p <0.0001 *vs* TNF*α*), suggesting that the treatment effectively limits NF-κB overexpression.

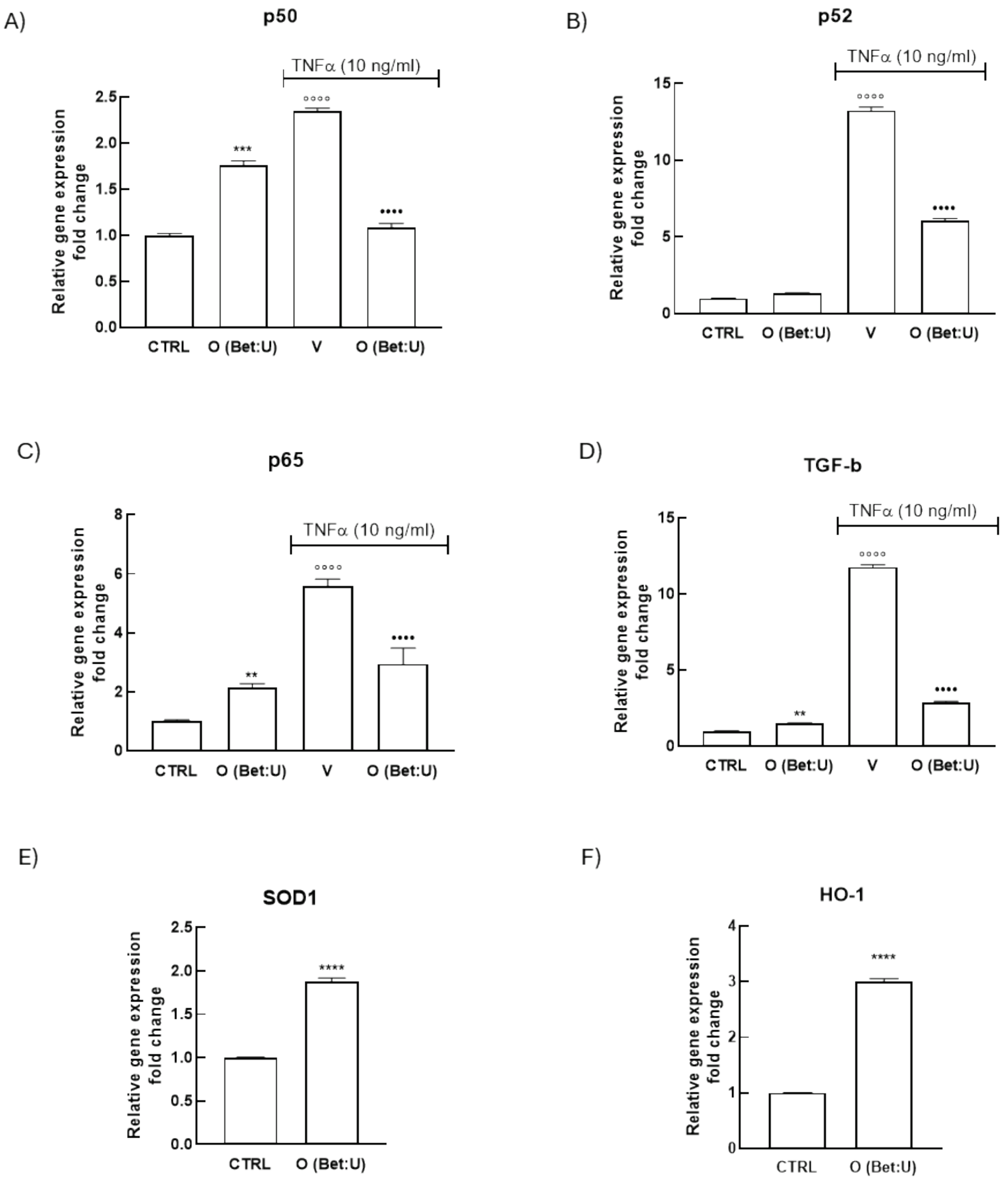

Figure S2: Effect of O$_{(Bet:U)}$ treatment on gene expression in HaCaT cells exposed to TNF-α. HaCaT cells were pretreated with O$_{(Bet:U)}$ (1:100, *v/v*) for 24 h and subsequently exposed to TNFα (10 ng/mL) for 16 h. Gene expression of p50 (**A**), p52 (**B**), p65 (**C**), TGF-β1 (**D**), SOD1 (**E**), and HO-1 (**F**) was analyzed by RT-PCR. Relative mRNA levels were calculated using the 2^−ΔΔCt method, with RPL13A as the reference gene. Results are expressed as means ± SEM of three independent experiments, each performed in triplicate. Significance was determined using one-way ANOVA and Dunnett's test. ** p <0.01, *** p <0.001 *vs.* Ctrl (control, untreated cells); •••• *p <0.0001 vs.* TNF-α-treated cells; °°°° p <0.0001 TNF-α-treated cells *vs.* Ctrl.

While a mild NF-κB activation can protect keratinocytes from apoptosis and support differentiation, chronic or excessive activation results in proliferative arrest and impaired wound repair [5]. By dampening but not abolishing NF-κB activity, O$_{(Bet:U)}$ likely preserves the transcriptional levels required for cell survival and migration, while preventing excessive expression that sustains prolonged inflammation, anti-apoptotic signaling, and uncontrolled proliferation [5].

Interestingly, TGF-β, a cytokine essential for wound closure and keratinocyte-fibroblast crosstalk [6], was upregulated by O$_{(Bet:U)}$ (p <0.01) (Figure S2, D) but was dramatically induced by TNFα (p <0.0001). Pretreatment

with $O_{(Bet:U)}$ markedly reduced this overexpression (p <0.0001), maintaining TGF-β level within a pro-healing yet non-fibrotic range. Such modulation is consistent with the dual nature of NF-κB in keratinocytes, where transient activation promotes survival and differentiation, but chronic overstimulation delays re-epithelialization and tissue remodeling.

In addition, treatment with $O_{(Bet:U)}$ significantly enhanced the expression of the antioxidant genes SOD1 and HO-1 (p <0.0001). This pattern suggests a robust activation of the Nrf2/ARE axis, which has been associated with improved wound closure, balanced ROS signalling, and prevention of chronic wound phenotypes [5,7]. Moreover, it has been shown that SOD1-deficient mice develop spontaneous skin lesions and display markedly delayed wound closure due to excessive oxidative stress and impaired keratinocyte migration [8]. Therefore, the up-regulation of SOD1 observed upon $O_{(Bet:U)}$ treatment may represent a crucial adaptive mechanism that enhances the antioxidant defense system and improves wound healing.

Overall, $O_{(Bet:U)}$ appears to orchestrate a balanced response, up-regulating Nrf2-dependent antioxidant defenses while mitigating excessive NF-κB/TGF-β activation. This synergy supports the improved wound-healing observed, where moderate IL-6/IL-8 release supports keratinocyte migration and controlled tissue regeneration.

References

1. Novotná, R.; Škařupová, D.; Hanyk, J.; Ulrichová, J.; Křen, V.; Bojarová, P.; Brodsky, K.; Vostálová, J.; Franková, J. Hesperidin, Hesperetin, Rutinose, and Rhamnose Act as Skin Anti-Aging Agents. *Molecules* **2023**, *28*, 1728, doi:10.3390/molecules28041728.
2. Cannavacciuolo, C.; Pagliari, S.; Frigerio, J.; Giustra, C.M.; Labra, M.; Campone, L. Natural Deep Eutectic Solvents (NADESs) Combined with Sustainable Extraction Techniques: A Review of the Green Chemistry Approach in Food Analysis. *Foods* **2022**, *12*, 56, doi:10.3390/foods12010056.
3. Calabria, D.; Guardigli, M.; Mirasoli, M.; Punzo, A.; Porru, E.; Zangheri, M.; Simoni, P.; Pagnotta, E.; Ugolini, L.; Lazzeri, L.; et al. Selective Chemiluminescent TURN-ON Quantitative Bioassay and Imaging of Intracellular Hydrogen Peroxide in Human Living Cells. *Anal Biochem* **2020**, *600*, 113760, doi:10.1016/j.ab.2020.113760.
4. Cao, G.; Alessio, H.M.; Cutler, R.G. Oxygen-Radical Absorbance Capacity Assay for Antioxidants. *Free Radic Biol Med* **1993**, *14*, 303–311, doi:10.1016/0891-5849(93)90027-R.
5. Ambrozova, N.; Ulrichova, J.; Galandakova, A. Models for the Study of Skin Wound Healing. The Role of Nrf2 and NF-KB. *Biomedical Papers* **2017**, *161*, 1–13, doi:10.5507/bp.2016.063.
6. Amento, E.P.; Beck, L.S. TGF-β and Wound Healing. *Ciba Found Symp* **2007**, *157*, 115-23, doi:10.1002/9780470514061.ch8.
7. Süntar, I.; Çetinkaya, S.; Panieri, E.; Saha, S.; Buttari, B.; Profumo, E.; Saso, L. Regulatory Role of Nrf2 Signaling Pathway in Wound Healing Process. *Molecules* **2021**, *26*, 2424, doi:10.3390/molecules26092424.
8. Iuchi, Y.; Roy, D.; Okada, F.; Kibe, N.; Tsunoda, S.; Suzuki, S.; Takahashi, M.; Yokoyama, H.; Yoshitake, J.; Kondo, S.; et al. Spontaneous Skin Damage and Delayed Wound Healing in SOD1-Deficient Mice. *Mol Cell Biochem* **2010**, *341*, 181–194, doi:10.1007/s11010-010-0449-y.
